# CircTBCK protects against osteoarthritis by regulating extracellular matrix and autophagy

**DOI:** 10.1007/s13577-025-01186-y

**Published:** 2025-02-25

**Authors:** Wei Wang, Yuzhe Sun, Peng Tang, Rui Zhang, Yufeng Jiang, Hongwei Min, Chen Gao

**Affiliations:** 1 Wenzhou Medical University, Wenzhou, Zhejiang China; 2 China Rehabilitation Science Institute, Beijing, China; 3 Department of Orthopedics and Rehabilitation, Beijing Bo’ai Hospital, China Rehabilitation Research Center, Beijing, China; 4 School of Rehabilitation, Capital Medical University, Beijing, China; 5 Beijing Key Laboratory of Neural Injury and Rehabilitation, Beijing, 100068 China

**Keywords:** CircRNA, CircTBCK, Osteoarthritis, Autophagy, ECM, QRT-PCR

## Abstract

Osteoarthritis (OA) is a widespread chronic bone and joint disease for which there is currently no effective preventive or therapeutic treatment. Accumulating evidence indicates that circular RNAs (circRNAs), a class of noncoding RNAs, play critical roles in OA. Therefore, in this study, we aimed to reveal an unexplored circTBCK and elucidate its mechanism of action in the pathological process of OA. The different expression of circTBCK was obtained both in vitro and in vivo. In the in vivo model, mice were induced via destabilization of the medial meniscus (DMM) surgery, while in vitro model, mouse cells like primary chondrocytes of newborn mice and ATDC5 cell line were treated with IL-1β treatment (10 ng/mL for 24 h). The level of circTBCK was examined by quantitative real-time polymerase chain reaction (qRT-PCR). After circTBCK was overexpressed or knocked down, IL-1β treatment was performed, and then, chondrocyte viability was detected via a Cell Counting Kit-8 (CCK-8) assay at 0, 24, 48, or 72 h. To assess type II collagen (Collagen II) expression, immunofluorescence (IF) analysis was used. The levels of mRNAs and proteins related to proliferation, the extracellular matrix (ECM) and autophagy were determined by qRT-PCR and Western blotting. Compared with OA treatment, primary chondrocytes with treatment of both circTBCK overexpression and IL-1βincreased the expression of anabolic factors—Collagen II and SRY-box transcription factor 9 (SOX9), proliferation-related molecules—Ki-67 and proliferating cell nuclear antigen (PCNA), and autophagy-related molecules—Microtubule-associated protein 1 light chain 3 (LC3), B-cell lymphoma 1 (Bcl1), and autophagy-related 5 (Atg5) and decreased Sequestosome 1 (SQSTM1 or P62). In contrast, knockdown of circTBCK aggravated the chondrocyte degeneration induced by IL-1β. Overall, our findings suggest that circTBCK, an unexplored circRNA, could regulate autophagy, proliferation, and the extracellular matrix (ECM) to mitigate the development of OA, suggesting a possible target for OA prevention and therapy.

**Supplementary Information:**

The online version contains supplementary material available at 10.1007/s13577-025-01186-y.

## Introduction

Osteoarthritis (OA) is a common chronic orthopedic disease that primarily affects middle-aged and elderly individuals and is characterized by the degeneration of joint cartilage, synovial hyperplasia, inflammation, subchondral bone destruction, and osteophyte formation [1]. Current strategies for treating OA are largely palliative and reactive, posing challenges for identifying early-stage OA, taking proactive and preventive actions, and delaying or reversing disease progression [2]. Cartilage degeneration is the main pathological feature of OA, with chondrocytes, the only cell type in articular cartilage, playing a crucial role in its progression. Numerous studies have shown that the abnormal biological functions of chondrocytes, including proliferation, apoptosis, extracellular matrix (ECM) formation, autophagy, and inflammation, are causally related to OA onset [3].

Circular RNAs (circRNAs) are a class of noncoding RNAs widely present in eukaryotic cells. They can act as miRNA sponges (competing endogenous RNA, ceRNA), regulate gene transcription, interact with proteins (RNA-binding protein, RBP), serve as protein scaffolds [4], and facilitate protein translation, thereby performing various biological functions [5]. With advancements in high-throughput technologies, an increasing number of circRNAs have been identified, and their relationships with diseases have been explored. Dysfunction of certain circRNAs plays a significant regulatory role in disease occurrence and progression, making them ideal biomarkers for early diagnosis, prognosis, and therapeutic response monitoring of various diseases, including tumors [6], cardiovascular diseases [7], neurological disorders [5], diabetes [8], skin injuries [9], and bone-related diseases [10].

Currently, many circRNAs have been proven to be differentially expressed in various pathological states of osteoarthritis. Zhou et al. reported significant upregulation of circRNA-33186 in an OA mouse model and a cell model [11]. Shen et al. discovered that circSERPINE2 regulates apoptosis and ECM anabolism via the miR-1271-ERG pathway [12]. Wu et al. suggested that decreased expression of circPDE4D promoted aggrecan loss and the upregulation of matrix catabolic enzymes [13].Given the association between circRNAs and the occurrence and development of OA, along with their high stability and tissue specificity [14], circRNAs could potentially serve as early diagnostic and prognostic biomarkers for OA.

However, research on this topic is still limited, making it urgent to elucidate more roles of circRNAs in OA and explore their underlying mechanisms in depth. This study aimed to uncover the role of an unexplored circRNA and circTBCK, and elucidate its mechanism of action in the pathological process of OA, potentially offering a new prevention and treatment target.

## Materials and methods

### Bioinformatics analysis

Using “osteoarthritis” and “circRNA” as keywords in the Gene Expression Omnibus (GEO) database, the dataset GSE178724 containing information on OA and control cartilage was retrieved. Differentially expressed circRNAs (DEGs) were screened via GEO2R (http://www.ncbi.nlm.nih.gov/geo/geo2r/) analysis with the following criteria: *p* value < 0.05 and |log2-fold change (FC)|> 1. Two subgroups of DEGs were identified: downregulated DEGs and upregulated DEGs. Volcano plots and heatmaps created by the Ouyi Cloud Platform (OE Biotech Co., China) were used for visualization.

### Chondrocyte extraction, culture, and treatment

Using previously outlined techniques, primary chondrocytes were isolated from the knee articular cartilage of newborn mice that were 5 days old [15]. The articular chondrocytes, isolated from five mice, were collectively cultured in a single flask. These mice were purchased from SPF Biotechnology Co., Ltd. (Beijing, China). In particular, primary chondrocytes were isolated from the knee joints by dissecting the tibial plateaus and femoral condyles. The separated cartilage was subsequently digested with 0.25% trypsin (including 0.02% EDTA) for 1 h and then with 0.2% type II collagenase for 8–16 h. To acquire primary chondrocytes, the digest was filtered through a 70 µm pore size mesh. ATDC5 [16] cells are a widely used cell line for studying cartilage- and bone-related diseases in vitro. These ATDC5 cells used in this study were obtained from GuangZhou Jennio Biotech Co., Ltd. (Guangzhou, China).

Both primary chondrocytes and ATDC5 cells were maintained in DMEM/F12 (GIBCO, USA) supplemented with 10% fetal bovine serum (GIBCO, USA) and 1% penicillin‒streptomycin (GIBCO, USA) and were cultured in an incubator at 37 °C with 5% CO_2_. Notably, ATDC5 cells were induced with insulin–transferrin–selenium (ITS) (Gibco) for 14 days before further experiments were performed.

To investigate the expression level of circTBCK in OA chondrocytes, IL-1β (10 ng/ml, PeproTech, Thermo Fisher Scientific, USA) was applied for a duration of 24 h. To determine the function of circTBCK in vitro, cells were pre-exposed with either small-interfering RNA (siRNA) targeting circTBCK (si-circTBCK) or lentivirus-overexpressing circTBCK (Lv-circTBCK) before IL-1β treatment. A Lipofectamine 3000 siRNA transfection system (Thermo Fisher Scientific, USA) was used to help siRNAs (GenePharma, China) transfect into the cells. Lv-circTBCK (HanBio, China) was added to the cell culture medium directly.

When investigating autophagic flux, we randomly divided primary chondrocytes into four groups: Lv-NC + IL-1β group, Lv-circTBCK + IL-1β group, Lv-NC + IL-1β + CQ group, and Lv-circTBCK + IL-1β + CQ group. First, cells in each group were transfected with Lv-NC or Lv-circTBCK for 24 h, followed by the addition of IL-1β (10 ng/ml) with or without chloroquine (CQ, 50 μM, MedChemExpress, MCE, China). After 24 h of incubation, we assessed the relevant indicators.

### RNA extraction, reverse transcription, and quantitative real-time PCR (qRT-PCR) analysis

TRIzol® reagent (Invitrogen, USA) was used to lyse the samples. After extraction, total RNA was analyzed via a NanoDrop system (Thermo Fisher, USA). Then, cDNA was generated using the FastKing RT Kit (with gDNase) (TIANGEN Biotech, China). SuperReal PreMix Plus (SYBR Green) (TIANGEN Biotech, China) was utilized in qRT-PCR experiments using QuantStudio3 (Thermo Fisher Scientific), and cDNA was extracted from every sample. The results were normalized to those of control samples and standardized to those of housekeeping genes, such as GAPDH, actin beta (ACTB), MALAT1, and U6. Supplementary Table S1 contains the specific primers that were utilized in this investigation (Sangon Biotech, China). To assess gene expression, the 2^-ΔΔCt^ relative fold change was computed.

### Western blotting

The total proteins were isolated using RIPA lysis buffer (Beyotime Biotechnology, China) supplemented with phenylmethyl sulfonyl fluoride, protease inhibitor cocktails, and phosphatase inhibitors (Servicebio, China). The protein concentration was determined via a BCA kit (Beyotime Biotechnology, China). Then, equivalent amounts of protein were separated via sodium dodecyl sulfate‒polyacrylamide gel electrophoresis (SDS‒PAGE) and transferred onto PVDF membranes (Millipore, USA). The membranes were blocked with protein-free quick blocking solution (Servicebio, China) for 15 min, and then, primary antibodies were added and incubated for an additional night at 4 °C (Supplementary Table S2). HRP-conjugated secondary antibodies (1:3000, Servicebio, China) were then applied at room temperature for 1 h. After washing, signals were detected using ChemiDoc MP (Bio-Rad Laboratories, USA) and analyzed with Image LabTM (Bio-Rad Laboratories).

### Cell proliferation assay

A Cell Counting Kit-8 (CCK-8) was utilized to assess the impact of circTBCK on chondrocyte proliferation. For the CCK-8assay, chondrocytes were transfected with siRNA or lentivirus for 24 h and then seeded into 96-well plates at a density of 2 × 10^3^ cells per well. The following day, IL-1β was added, and the cells were cultured for 0, 24, 48, or 72 h. Cell proliferation was assessed using CCK-8, and the absorbance at 450 nm was measured via EnSpire (PerkinElmer, USA).

### Immunofluorescence analysis

Sterile cell slides were carefully inserted into a 6-well plate, and 3 × 10^4^ chondrocytes were seeded in each well. When the cells reached 60–70%, the chondrocyte slides were fixed at room temperature with 4% paraformaldehyde fixative, permeabilized with 0.5% Triton X-100, and blocked with 5% bovine serum albumin (BSA) buffer. They were subsequently incubated in a wet box at 4 °C overnight with primary antibodies against type II collagen (Collagen II) (dilution 1:100; Abcam, UK). The secondary antibodies (BEIJING ZHONGSHAN GOLDEN BRIDGE BIOTECHNOLOGY CO., LTD., China) were conjugated to the primary antibodies for one hour at RT in the dark. After that, DAPI staining and anti-fluorescence quenching tablets were used to seal the slides. A Laser Confocal microscope (Nikon, Japan) was used to take digital fluorescence images at × 20 magnification. Image software (Media Cybernetics, USA) was used to evaluate the measurements.

### Nuclear-cytoplasmic fractionation

According to the manufacturer's instructions, PARIS Kit (Invitrogen, USA) was used to isolate cytoplasmic RNA and nuclear RNA. To put it briefly, chondrocytes were lysed with cell fractionation buffer, and then, a centrifuge was used to separate the two cell components. After the supernatant was transferred to fresh RNase-free tubes, the remaining lysate was washed with cell fractionation buffer. Cell disruption buffer was used to divide the nuclei. The above lysate and the supernatant were mixed with a 2X lysis binding solution, and an equal volume of ethanol was added through a filter cartridge. The solution was then used to wash the samples. After being eluted, the cytoplasmic and nuclear RNA were reverse transcribed into cDNA and analyzed by qRT-PCR.

### RNase R treatment

To detect the stability of mRNAs and circRNAs, RNase R detection was performed. Total RNA (5 µg) was treated with or without 3 U/µg RNase R (Beyotime Biotechnology, China) at 37 °C for 20 min, followed by inactivation at 70 °C for 10 min. QRT‒PCR was then used.

### RNA fluorescence in situ hybridization

CY3-labeled circTBCK probes were designed and synthesized by Servicebio (Wuhan, China). The cell nuclei were labeled with 4’,6-diamidino-2-phenylindole (DAPI) (Servicebio, China). The probe signals were determined via a FISH Kit (Servicebio, China) according to the manufacturer’s guidelines. Images were obtained via a laser confocal microscope (Nikon, Japan).

### Animal experiments

All animal experiments were approved by the Ethical Committee of Animal Experiments and Experiments Animal Welfare Capital Medical University and were performed in accordance with the committee’s guidelines. Eight-week-old male C57BL/6 J mice (*n* = 8; mean body weight = 25 g) were purchased from SPF Biotechnology Co., Ltd. (Beijing, China) and randomly divided into two groups: the posttraumatic OA model group (*n* = 4) and the control group (*n* = 4).

The allocation of mice was concealed using a computer-generated randomization system by an independent assistant who remained uninvolved in the experimental procedures. This assistant assigned each mouse a random code, ensuring their random allocation into either the model or control group. The experimenters were kept blinded to these group assignments throughout the study, only being informed of the specific surgical procedures at the time of surgery. After the surgeries were completed, the blinding continued up to the data analysis phase.

Destabilization of the medial meniscus (DMM) surgery was done on the mice in the positive control group to establish a posttraumatic OA model, as previously described [17]. In short, following anesthesia, the right knee joints of the mice were exposed via a medial capsular incision. The ligaments linked to the tibial plateau were then released by severing the medial meniscotibial ligament (MMLT) with microsurgical scissors, which destabilized the medial meniscus. Ultimately, the skin was closed, and the wound was stitched. In parallel, control animals underwent a fake procedure that involved just incising the medial knee joint capsule.

The mice were randomly assigned to separate cages within the animal facility to prevent potential bias. All animals were housed under standard conditions, with continuous access to food and water ad libitum. Throughout the experiment, researchers remained blinded to the treatment groups.

The mice were euthanized 8 weeks after surgery, and their knee cartilage was removed and subjected to imaging and biochemical analysis. Researchers remained blinded to the group allocations during the assessment process. To guarantee an unbiased evaluation, animals were selected for outcome assessment using a random number table, which was generated by a computer system.

### Microcomputed tomography (micro-CT) scanning

Micro-CT scanning was done using a micro-CT scanner (Skyscan 1276, Bruker, Germany). Micro-CT analysis software (CTvox, SkyScan) was used to run the three-dimensional reconstruction and image viewing of the knee joint.

### Statistical analyses

At least three separate biological replicates or three repeated measurements were used to gather all of the data. The experimental data are presented as the means ± standard deviations. The distribution of the data was tested with the Shapiro‒Wilk test, and the equality of variances was tested using Levene’s test. The statistical significance of differences between two groups was determined via Student’s t test. Multiple group comparisons were determined by one-way ANOVA followed by the Bonferroni correction unless otherwise indicated. Variations were deemed statistically significant when P values (two-sided) were less than 0.05. SPSS 25 (SPSS, Inc., Chicago, IL, USA) and GraphPad Prism 8 (GraphPad Software, San Diego, CA, USA) were used to analyze the data.

## Results

### Identification of DEGs

**Fig. 1:**
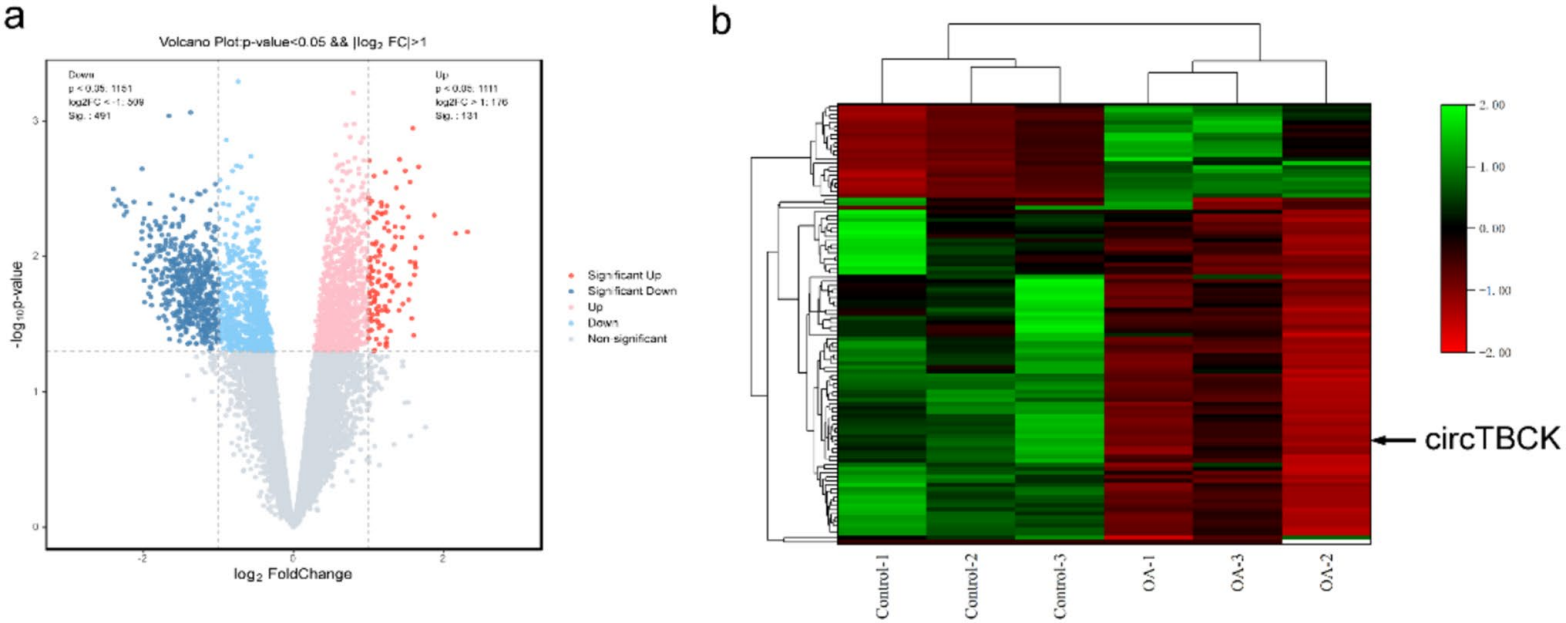
CircTBCK differential expression in GSE178724: **a** Volcano plots and **b** heat map of differentially expressed circRNAs between OA and control cartilage samples

Using the criteria given above, a total of 622 DEGs were found in GSE178724. All the upregulated and downregulated DEGs in arthritic and nonarthritic cartilage are displayed in volcano plots (Fig. 1a). A portion of these DEGs’ expression patterns are displayed in heatmaps (Fig. 1b). We arranged |logFC| in descending order on the basis of *P* < 0.05 and selected the top 20 upregulated and downregulated genes, which were referred to as candidate DEGs.

### CircTBCK expression is relatively low in OA tissues and cells

**Fig. 2:**
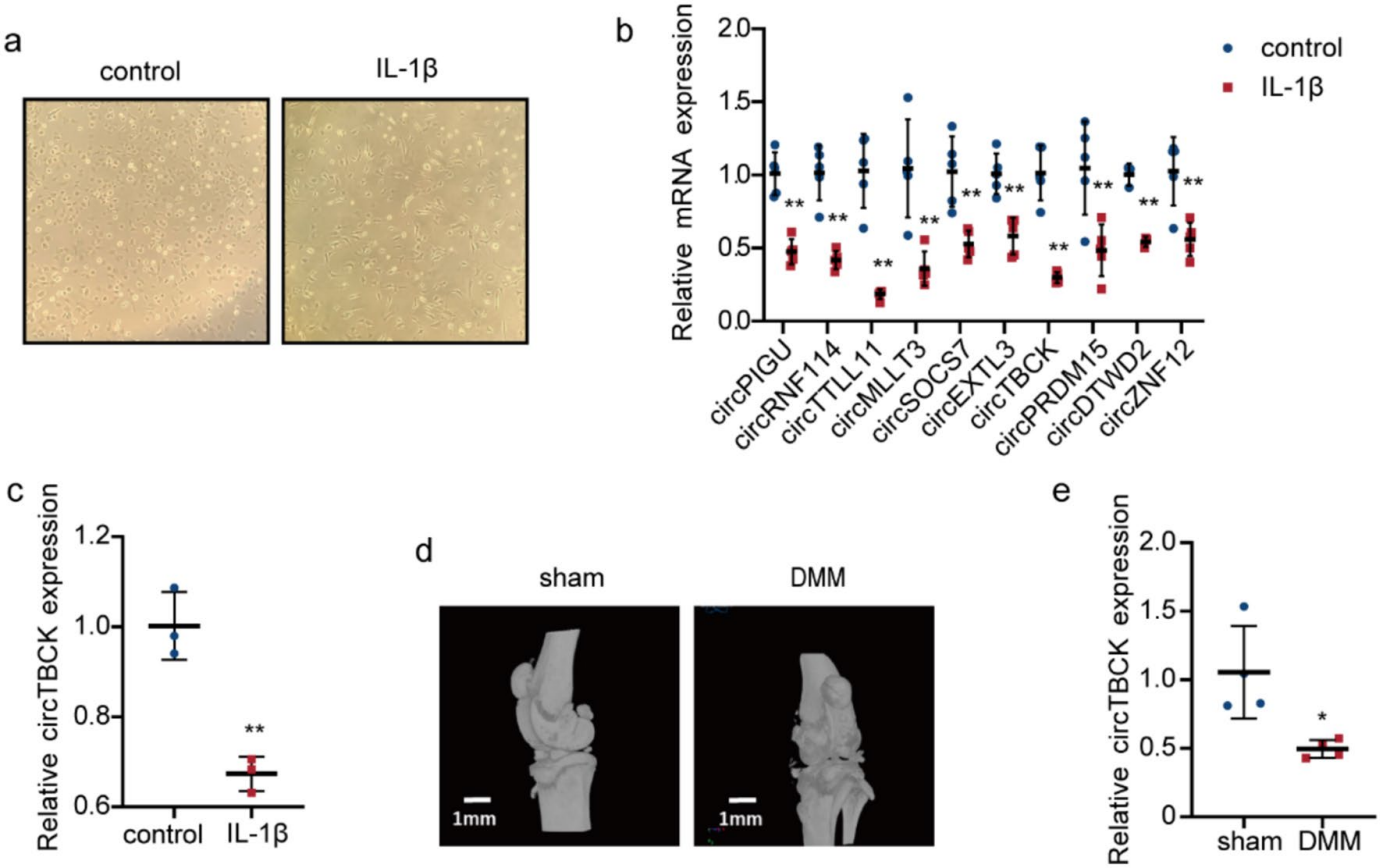
CircTBCK is downregulation in OA tissues and cells: **a** Images of primary chondrocytes with or without IL-1β treatment for 24 h, under inverted microscope. **b** Expression of candidate DEGs was measured by qRT-PCR after primary chondrocytes were treating with IL-1β. *n* = 5 per group for all while *n* = 3 for circDTWD2 detection. **p < 0.01. **c** CircTBCK expression level of ATDC5 by qRT-PCR after treating with IL-1β. *n* = 5 per group. **p < 0.01. **d** micro‐CT imaging for morphological structure in the knee of OA mice at 8 weeks after DMM or sham surgery. *n* = 4. Scale bar = 1 mm. **e** The circTBCK expression measured by qRT‐PCR is lower in mouse OA cartilage than control cartilage. *n* = 4. *p < 0.05

To validate the expression of candidate DEGs in OA, in vitro and in vivo OA models were established, and qRT-PCR was carried out. In the in vitro model, after 24 h of treatment with IL-1β, the morphology of the chondrocytes became elongated, and the cell state was affected (Fig. 2a). qRT-PCR revealed that the expression levels of circPIGU, circRNF114, circTTLL11, circMLLT3, circSOCS7, circEXTL3, circTBCK, circPRDM15, circDTWD2, and circZNF12 were downregulated to a statistically significant degree compared with control group, as shown in Fig. 2b. We are very interested in circTBCK among these candidate DEGs. We then performed an ATDC5 cell experiment and showed the same results (Fig. 2c).

Next, we prepared a mouse DMM model, and through in vivo micro-CT detection, which was accompanied by the formation of osteophytes, the joint space of OA model mice significantly increased (Fig. 2d). These observations were consistent with the description of Zhou [18]. To ensure comparability between the intervention and control groups at baseline, we analyzed prognostic factors such as age, weight, and sex. These characteristics confirmed that the groups were comparable at the start of the experiment. Eight weeks after DMM surgery, we harvested articular cartilage from the distal femur and tibial plateau for qRT-PCR to validate the expression of circTBCK. As predicted, it was markedly downregulated in OA cartilage (Fig. 2e). We meticulously monitored the outcome data, confirming no attrition or exclusions, thereby ensuring the completeness of our data. Additionally, we performed a review of selective outcome reporting, verifying that all primary outcomes had been pre-registered and consistently reported.

### Characteristics and distribution of CircTBCK

The same host gene can form circRNAs and usually has corresponding homologous mRNAs. Moreover, the backsplice of circRNA and the variable splicing of mRNA share a nonspecific splicing mechanism. However, due to the fact that circRNA is a type of RNA molecule without free 3’ poly A tails or 5’ caps and forms a circular structure through covalent bonds, its structure is relatively stable and not easily hydrolyzed by enzymes, and there is a backstage junction (BSJ), which is the two main difference between circRNA and linear RNA. Using the previously described methodology [5], we followed a number of measures to rule out these possibilities to confirm that the study target was a circRNA rather than an mRNA.

**Fig. 3:**
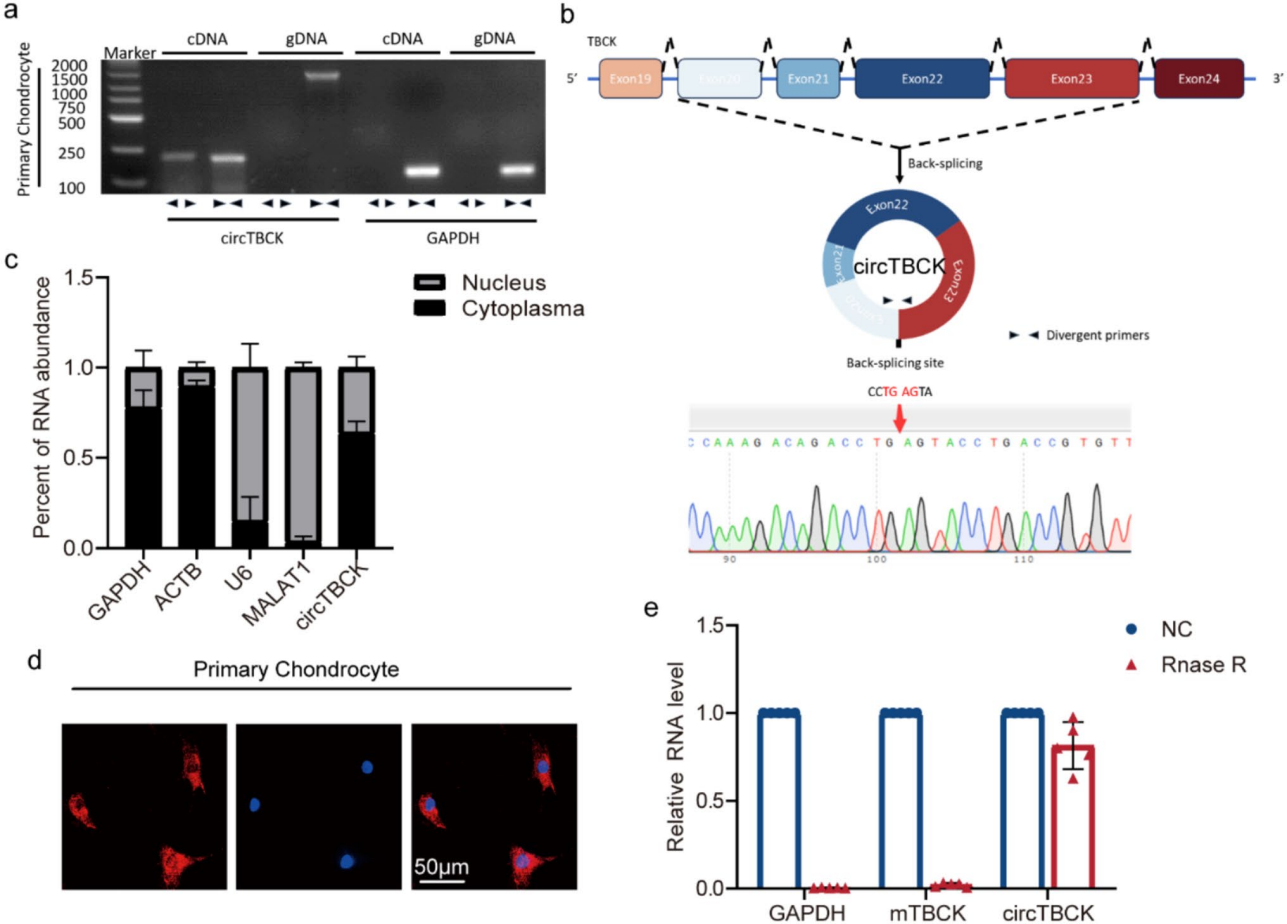
Characteristics and distribution of circTBCK. **a** Using Convergent and Divergent primers, the cDNA and gDNA amplification products of primary chondrocytes were separately subjected to agarose gel electrophoresis. **b** Schematic illustration showing TBCK exons 20–23 circularisation to form circTBCK (black arrow). Sanger sequencing was performed. Red arrow represents head-to-tail circTBCK splicing sites. **c** Subcellular distribution of circTBCK was detected by Nuclear–Cytoplasmic fractionation assay in primary chondrocytes. *n* = 5.MALAT1 and U6 acted as nuclear controls, whereas GAPDH and ACTB acted as cytoplasm controls. **d** RNA FISH revealed the predominant localisation of circTBCK in the cytoplasm. circTBCK probes were labeled with Cy-3. Nuclei were stained with DAPI. Scale bar, 50 µm. **e** The expression of circTBCK and linear TBCK mRNA in primary chondrocytes treated with or without RNase R was detected by qRT-PCR. *n* = 5

First, aiming at cDNA and genomic DNA (gDNA), we designed and synthesized convergent primers and divergent primers to amplify TBCK mRNA and circTBCK, respectively. CircTBCK was amplified by divergent primers from cDNA but not from gDNA (Fig. 3a). Second, Sanger sequencing supported the circTBCK splicing sequence (Fig. 3b). Third, following RNase R treatment, we verified that circTBCK was resistant to RNase R, while the TBCK mRNA level dramatically decreased (Fig. 3e). Moreover, the cellular localization of circTBCK is closely related to its biological function. Therefore, FISH and qRT-PCR analyses for nuclear‒cytoplasmic fractionation were performed and indicated the phenomenon that circTBCK had a higher abundant in cytoplasmic than in the nucleus (Fig. 3c, d).

### Overexpression of circTBCK alleviates the degeneration of OA chondrocytes

**Fig. 4:**
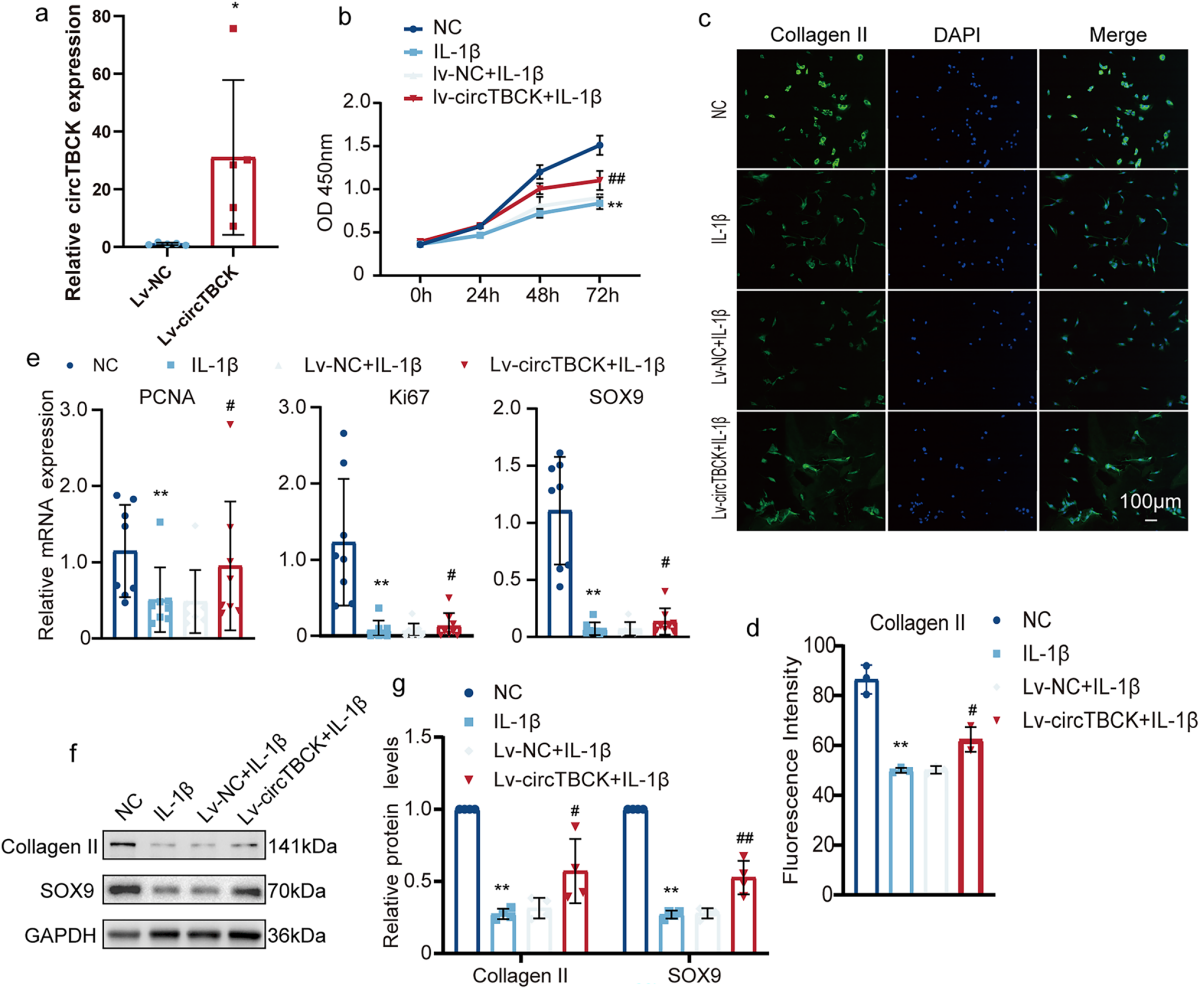
CircTBCK overexpression recovered the proliferation and anabolism of ECM in OA chondrocytes: **a** Chondrocytes were transfected with circTBCK lentivirus or control virus. After 48 h of transfection, the expression level of circTBCK was measured by qRT-PCR and normalized to GAPDH level. *n* = 5. *p < 0.05 . **b** Cell proliferation determined by CCK-8 assay. *n* = 4. **p < 0.01 compared with NC group; ##*p* < 0.01 compared with IL-1βgroup. **c–d** Representative photomicrographs and IF of Collagen II in chondrocytes with or without circTBCK lentivirus transfection and with or without IL-1β treatment. *n* = 3. Scale bar, 100 µm. **p < 0.01, #*p* < 0.05. **e** Relative expression of PCNA, Ki-67, and SOX9 were measured by qRT-PCR. *n* = 8. **p < 0.01 compared with NC group; #*p* < 0.05 compared with IL-1β group. **f–g** Western blot analysis of Collagen II and SOX9 when circTBCK was upregulated in OA chondrocytes. The optical density analysis was performed from the results of four independent experiments of western blot samples. **p < 0.01 compared with NC group; #*p* < 0.05, ##*p* < 0.01 compared with IL-1β group

Next, we conducted trials on function repair. Chondrocytes were transfected with circTBCK lentivirus or control virus. After 48 h, circTBCK expression was measured via qRT-PCR, which revealed that circTBCK was overexpressed (Fig. 4a). After being pre-exposed to Lv-circTBCK for 24 h, the chondrocytes were treated with IL-1β for 24 h. Overexpressing circTBCK in OA chondrocytes boosted cell viability relative to that in the IL-1β group, as demonstrated by a CCK-8 experiment (Fig. 4b). QRT‒PCR detection of Ki67 and proliferating cell nuclear antigen (PCNA) produced consistent results (Fig. 4e). In addition, circTBCK upregulation expression obviously ameliorated COL2A1 and SRY-box transcription factor 9 (SOX9) levels in cells, as evidenced by the IF (Fig. 4c–d), qRT-PCR (Fig. 2e) and western blot results (Fig. 4f‒ g). These data clearly indicate the effects of circTBCK on the proliferation and collagen formation of chondrocytes.

**Fig. 5:**
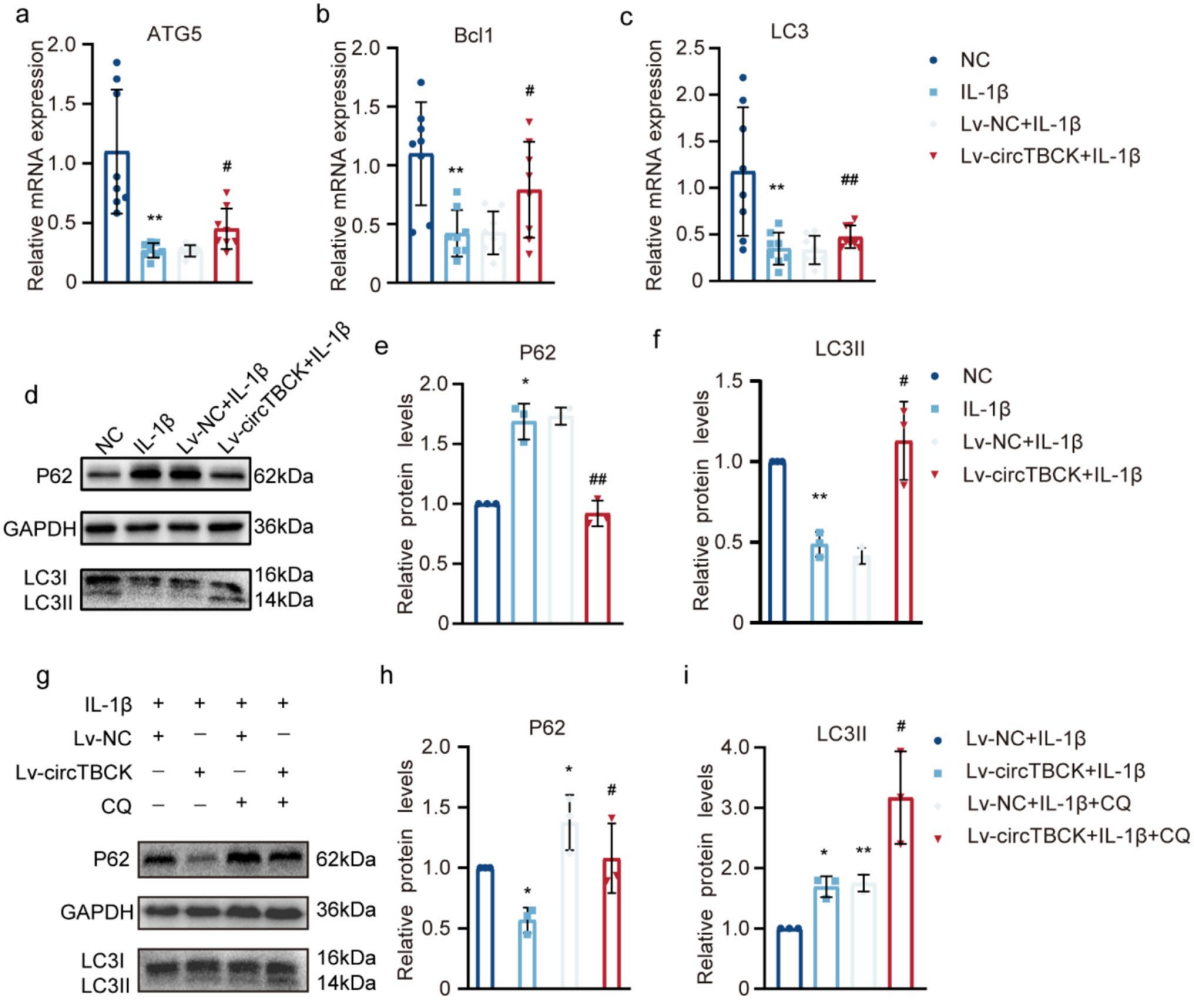
Effects of circTBCK overexpression on autophagy in IL-1β-induced chondrocytes **a-c** qRT-PCR detected the expression of ATG5, Bcl1, and LC3. *n* = 8. **p < 0.01 compared with NC group; #*p* < 0.05 ,##p＜0.01 compared with IL-1β group. **d–f** Western blot analyzed the protein levels of P62, LC3I, and LC3II. The optical density analysis was performed from independent experiments of western blot samples. *n* = 3.*p < 0.05, **p＜0.01 compared with NC group;#p＜0.05, ##*p* < 0.01 compared with IL-1β group. **g** Representative western blot of P62 and LC3 in OA primary chondrocytes pretreated with or without Lv-circTBCK in the presence or absence of CQ (50 μM) for 24 h. **h-i** Quantitative analysis of the protein levels of P62 (Fig. 5 h) and LC3II (Fig. 5i). n = 3. *p < 0.05 compared with Lv-NC + IL-1β group, **p < 0.01 compared with Lv-NC + IL-1β group, #*p* < 0.05 compared with Lv-circTBCK + IL-1β group

Moreover, in terms of autophagy, overexpression of circTBCK significantly ameliorated the expression of B-cell lymphoma 1 (Bcl1), microtubule-associated protein 1 light chain 3 (LC3), autophagy-related 5 (Atg5) and decreased Sequestosome 1 (SQSTM1 or P62) in OA chondrocytes, as shown by qRT-PCR (Fig. 5a‒c) and western blotting (Fig. 5d‒f).

It can be clearly inferred the result that overexpressing circTBCK could mitigate the degeneration of chondrocytes in terms of proliferation, ECM synthetic metabolism in OA chondrocytes. However, in terms of autophagy, we not only assessed the expression of autophagy-related proteins by regulating circTBCK, but also used Chloroquine (CQ) as an autophagy inhibitor to monitor changes in autophagic flux. As shown in Fig. 5g–i, compared to circTBCK overexpression alone, the protein expression levels of P62 and LC3 were significantly increased in OA cells treated with both circTBCK overexpression and CQ, indicating that circTBCK overexpression enhanced autophagic flux. These findings elucidated the relationship between circTBCK overexpression and autophagic levels in OA chondrocytes.

### Knocking down circTBCK exacerbates the degeneration of OA chondrocytes

**Fig. 6:**
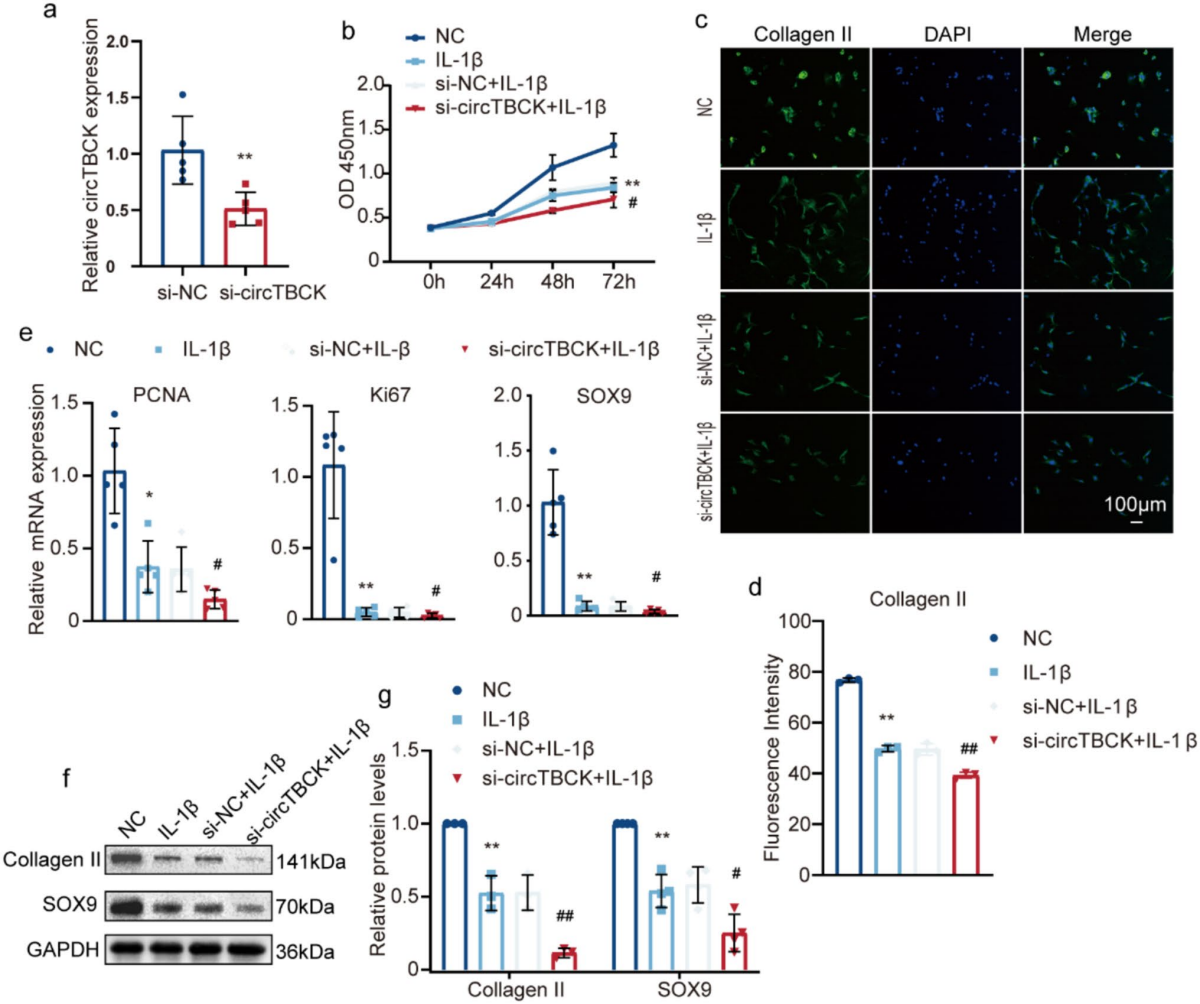
CircTBCK expression knockdown inhibited the proliferation and anabolism of ECM of chondrocytes. Chondrocytes were transfected with circTBCK siRNA or negative control siRNA at a final concentration of 20 nM for 48 h.c **a** The results of circTBCK expression knockdown by siRNA was measured by qRT-PCR and normalized to GAPDH level. *n* = 5. **p < 0.01. **b** CCK-8 assay detected the cell proliferation. *n* = 5. **p < 0.01 compared with NC group; #*p* < 0.05 compared with IL-1β group. **c-d** Representative photomicrographs and fluorescence intensity of IF of Collagen II in chondrocytes with or without si-TBCK transfection and with or without IL-1β treatment. *n* = 3. Scale bar, 100 µm. **p < 0.01, ##*p* < 0.01. **e** Relative expressions of PCNA, Ki-67, and SOX9 were tested by qRT-PCR. *n* = 5. *p < 0.05,**p < 0.01 compared with NC group; #*p* < 0.05 compared with IL-1β group. **f–g** Collagen II and SOX9 protein levels were analyzed by Western blot. And the optical density analysis was performed. *n* = 3. **p < 0.01 compared with NC group; #p＜0.05, ##p < 0.01compared with IL-1β group

**Fig. 7:**
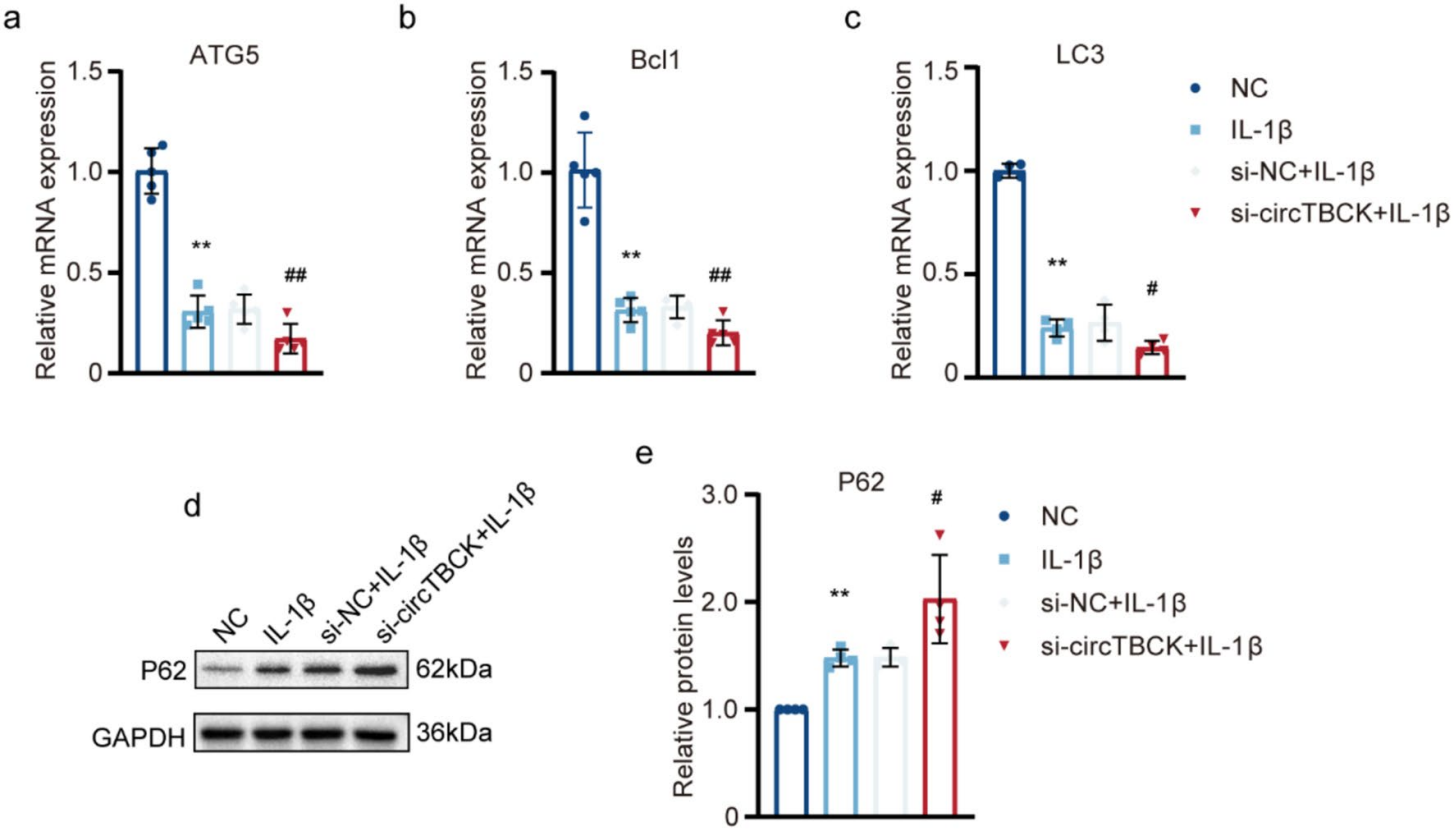
Effects of circTBCK knockdown on autophagy in IL-1β induced chondrocytes **a–c** qRT-PCR determined the expression of ATG5, Bcl1, and LC3.*n* = 5, **p < 0.01 compared with NC group; #*p* < 0.05, ##*p* < 0.01 compared with IL-1β group. **d–e** The protein levels of P62 were analyzed by Western blot. The optical density analysis was performed from four independent experiments of western blot samples. **p < 0.01 compared with NC group; #*p* < 0.05 compared with IL-1β group

To assess the influence of lower expression levels of circTBCK on cartilage degeneration, before IL-1β treatment, we transfected primary chondrocytes with circTBCK siRNAs. qRT-PCR was used to determine the interference efficiency (Fig. 6a). We then assessed the relationship between circTBCK and chondrocyte viability via a cell counting kit-8 (CCK-8) assay. The results showed that knockdown of circTBCK expression severely reduced OA chondrocyte viability (Fig. 6b). In addition, the inhibition of circTBCK significantly downregulated the expression of SOX9, PCNA, Ki-67, Bcl1, LC3, and ATG5 in OA chondrocytes, as revealed by qRT-PCR (Fig. 6e, 7a‒c), and attenuated the protein levels of Collagen II, SOX9, P62, and LC3II, as shown by western blotting (Fig. 6f‒ g, 7d‒e). Immunofluorescence further confirmed that circTBCK knockdown decreased the Collagen II level (Fig. 6c–d). These data clearly indicate the phenomenon that circTBCK knockdown exacerbates the degeneration of chondrocytes in terms of proliferation, collagen formation, and autophagy in OA chondrocytes.

## Discussion

Although efforts have been made in recent decades to elucidate the pathogenesis of this disease, the medical treatment of osteoarthritis still focuses only on alleviating the pain symptoms of osteoarthritis and joint arthroplasty [19]. Effective preventive and curative methods are lacking. Identifying reliable biomarkers for OA has remained a challenge [20]. Consequently, it is important to.

investigate the molecular processes driving the advancement of OA and to search for diagnostic and therapeutic targets for OA. Compared with linear RNA, circRNA is more abundant (approximately tenfold) and highly stable [21]. With the advancements in biotechnology and molecular medicine, artificial circRNAs have been developed as a unique class of vaccines for disease prevention and therapy [22]. Therefore, circRNAs have enormous potential as diagnostic and therapeutic targets.

Here, we first identified circTBCK as a differentially expressed circRNA through sequencing data. In addition, circTBCK is generated by back-splicing of the TBCK gene, the sequence of which is relatively conserved between humans and mice. Therefore, we used mice as an animal model. circTBCK is downregulated in the cartilage of OA model mice caused by DMM, as well as in primary chondrocytes and ATDC5 cells treated with IL-1β. The inverse relationship between circTBCK and chondrocyte changes suggested that circTBCK is likely associated with OA development. Furthermore, to investigate the function of circTBCK in the proautophagy response, proliferation regulation, and protection of ECM components from degradation, we carried out functional repair tests in vitro. Potential confounding factors, such as age, sex, and weight, of the experimental mice, which can influence OA progression, were controlled using age-matched, weight-matched male mice throughout our experiments.

The progressive degradation of cartilage tissue and abnormalities in chondrocytes are considered important factors in the occurrence of osteoarthritis [23]. Normal chondrocytes have very active metabolism and play a crucial role in maintaining the normal function of cartilage. After being stimulated by inflammatory factors, the proliferation of chondrocytes is inhibited, and they are prone to differentiate into fibroblast-like phenotypes [24]. In our study, compared with that of normal chondrocytes, the proliferation ability of OA chondrocytes decreased, and knocking down circTBCK aggravated this expression. In contrast, overexpression of circTBCK relieved these effects. qRT-PCR detection of the expression levels of proliferation markers, such as ki-67 and PCNA, yielded consistent results.

During normal cartilage tissue homeostasis, ECM synthesis, metabolism, and catabolism are in a dynamic equilibrium state. As a major component of the cartilage ECM, type II collagen plays an important role in maintaining the mechanical properties of articular cartilage and the stability of the ECM. In addition, COL2A1 is regulated by SOX9. Zhao et al. [25] reported that a new SOX9-binding site in intron 6 of the Col2a1 gene appears to be necessary for strong expression of Col2a1. According to our results, the expression levels of type II collagen and SOX9 in the chondrocytes of the model group decreased, but overexpression of circTBCK reversed this trend, indicating a positive correlation between circTBCK and the ECM.

Autophagy seems to be a protective mechanism that controls the balance and stability of the microenvironment within articular cartilage [26]. When chondrocytes are under various types of stress, autophagy helps them devour and recirculate macromolecules and organelles to adapt to changing environmental conditions [27]. However, as time progresses, autophagy activity decreases in osteoarthritis. To explore the effect of circTBCK on chondrocyte autophagy, we focused on four autophagy-related proteins: LC3, P62, Beclin 1, and Atg5. LC3 [28] is a specific marker of autophagosomes. During autophagy, cytoplasmic LC3-I is converted to the phospholipid-conjugated form of LC3-II and is continuously recruited into autophagic vacuoles [29]. P62 is a substrate of LC3, which is degraded during autophagosome–lysosome fusion and is negatively correlated with autophagy [30]. Beclin 1 plays a key role in autophagy by regulating the formation of autophagosomes [31]. As one of the most important autophagy-related proteins, autophagy-related gene 5 (Atg5) is a marker of autophagic activity [32]. In this study, after treatment with IL-1β (10 ng/ml) for 24 h, we detected a decrease in autophagy in chondrocytes, which was consistent with the results of Zhou et al. [33]. Furthermore, we detected a positive correlation between circTBCK and autophagy in articular chondrocytes. However, autophagy is a dynamic process, and monitoring autophagic flux is essential [34]. It typically requires the use of autophagy flux inhibitors by Western blotting. Chloroquine (CQ) is an autophagosome–lysosome fusion inhibitor, and the difference in the expression levels of LC3II and P62 in the presence or absence of CQ is used to reflect autophagic flux. If flux occurs, the expression levels of LC3II and P62 would be higher in the presence of CQ. As discussed earlier, we observed that circTBCK overexpression increased LC3II protein levels. To determine whether this increase represents autophagy induction and enhanced LC3 synthesis, or a reduction in flux and subsequent LC3 accumulation, we treated OA cells with circTBCK overexpression and used chloroquine to block the degradation of autophagosomes. We then measured LC3II and P62 levels to assess autophagic flux. Following CQ treatment, there was an increase in the protein expression levels of P62 and LC3II, suggesting that autophagic flux had been enhanced. These results indicated that circTBCK may participate in OA through autophagy.

Based on the information provided, we speculated that circTBCK played a significant role in the onset and progression of osteoarthritis (OA), and targeting circTBCK through gene therapy may introduce a novel treatment avenue for OA. Initially, adenoviruses (viral vectors) and plasmids (non-viral expression vectors) are the most commonly employed vectors in preclinical research. Traditionally, complementary sequence-mediated RNA circularization has been utilized to upregulate circRNA. Nevertheless, a vector incorporating upstream introns, downstream intron fragments, and circRNA circularization sequences has demonstrated superior circRNA amplification efficiency [5]. For instance, in vitro, specific circRNAs can be synthesized by cloning the target sequence into an artificial exon and subsequently transfecting the plasmid into cells [35]. Additionally, hydrogel-guided overexpression using recombinant adeno-associated virus vectors has been shown to repair articular cartilage defects [36]. Thus, circTBCK can be prepared in vitro and employed via adenovirus or plasmid vectors to enhance circTBCK expression in OA articular cartilage, potentially reversing cartilage degeneration. Moreover, extracellular vesicles have emerged as potent carriers, especially in the realm of delivering ncRNAs, owing to their nanoscale size, low immunogenicity, ability to penetrate biological membranes, and ease of storage [37].Yang et al. [38]reported that extracellular vesicle-mediated delivery of circSCMH1 could improve stroke prognosis in mice and monkeys. Similarly, extracellular vesicle-mediated circ_0001846 has been involved in modulating IL-1β-induced chondrocyte injury [39]. Therefore, employing extracellular vesicles to deliver therapeutic circRNAs to OA sites may represent an innovative cell-free therapy option for OA. Extracellular vesicles could be utilized to load circTBCK and transport it directly to articular chondrocytes to exert therapeutic effects. Given their circular structure, circRNA-based therapies are potentially safer, more stable, easier to manufacture, and more scalable than linear mRNA therapies [22]. Consequently, developing circRNA vaccines targeting circTBCK could hold substantial clinical promise.

Although we confirmed that circTBCK may exert protective effects by regulating OA chondrocyte proliferation, maintaining ECM stability, and regulating autophagy, there still remained some limitations and recommendations for future research:

First, the specific pathway through which circTBCK operates remains unclear. The signal transduction pathways involved in osteoarthritis are complex and interdependent [40]. Zhao et al. [25] revealed that circFOXO3 activates autophagy via the PI3K/AKT pathway. Shen et al. [41] reported that circPDE4B plays an important role in regulating p38 MAPK signaling in OA. Zhang et al. [42] reported that circMELK inhibits chondrocyte autophagy in OA by regulating NF-κB signaling. These findings imply that the pathophysiology of OA involves the signaling system in a significant way.

Second, circRNAs are not affected by RNA exonucleases and typically target miRNAs as mediators of autophagy. Owing to their abundant binding sites, high stability, and disease specificity, circRNAs are likely to function as ceRNAs [19] under different physiological and pathophysiological conditions. We found through in situ hybridization and cytoplasmic nuclear separation PCR experiments that circTBCK is distributed mainly in the cytoplasm; therefore, we speculate that circTBCK could target a certain miRNA by functioning as a sponge. We then predicted through a website that circTBCK has multiple miRNA-binding sites, but whether it binds to miRNAs and through which miRNAs it exerts biological effects is still worth further exploration.

Third, although this study primarily focuses on the effects of circTBCK on ECM regulation and autophagy, the potential influence of circTBCK on subchondral bone remodeling remains an intriguing avenue for future research. Given the known interactions between cartilage and subchondral bone in osteoarthritis, it is possible that circTBCK may contribute to the remodeling of subchondral bone through its regulatory effects on ECM and autophagy. Further studies are needed to investigate how circTBCK may impact the balance between osteoblastic and osteoclastic activity in the context of OA progression.

Fourth, the feasibility of implementing circTBCK in clinical practice appears promising, given its role in regulating OA cartilage in mice both in vivo and in vitro. However, as this is the inaugural report on circTBCK, there are no studies on its effects in other diseases or using different animal and cell models. Therefore, there is an urgent need for additional animal and cell models to validate circTBCK’s effects on osteoarthritis. Moreover, further validation through clinical trials is essential to establish the therapeutic efficacy and safety of targeting circTBCK gene therapy. Although there has been no prior research on circTBCK and potential side effects, including immune reactions, are unknown, careful monitoring of patient responses will be crucial.

In conclusion, our study revealed that circTBCK, a novel circRNA that likely plays an important role in OA progression, including aspects, such as autophagy, proliferation, and extracellular matrix (ECM). Targeted regulation of circTBCK may become an effective approach for the prevention and treatment of osteoarthritis. Although our study provides new options for OA treatment, further research is needed to validate and better understand the role of circTBCK in the pathophysiology of OA.

## Supplementary Information

Below is the link to the electronic supplementary material.

Supplementary file1 (PDF 849 KB)

Supplementary file2 (PDF 912 KB)

Supplementary file3 (XLSX 13 KB)

Supplementary file4 (PDF 108 KB)

Supplementary file5 (PDF 592 KB)

Supplementary file6 (PDF 646 KB)

Supplementary file7 (XLSX 12 KB)

Supplementary file8 (PDF 1009 KB)

Supplementary file9 (PDF 659 KB)

Supplementary file10 (PDF 642 KB)

Supplementary file11 (PDF 381 KB)

Supplementary file12 (XLSX 16 KB)

Supplementary file13 (PDF 715 KB)

## Data Availability

The datasets analyzed during the current study are available in the National Center for Biotechnology Information (National Center for Biotechnology Information (nih.gov)).
