# Supplementary material for: CircTBCK protects against osteoarthritis by regulating extracellular matrix and autophagy
Source: Hum Cell. 2025 Feb 25;38(2):60. doi: 10.1007/s13577-025-01186-y (PMC11860995; doi:10.1007/s13577-025-01186-y)
Supplement: Supplementary file 2 — Supplementary file2 (PDF 912 KB) [file 13577_2025_1186_MOESM2_ESM.pdf]

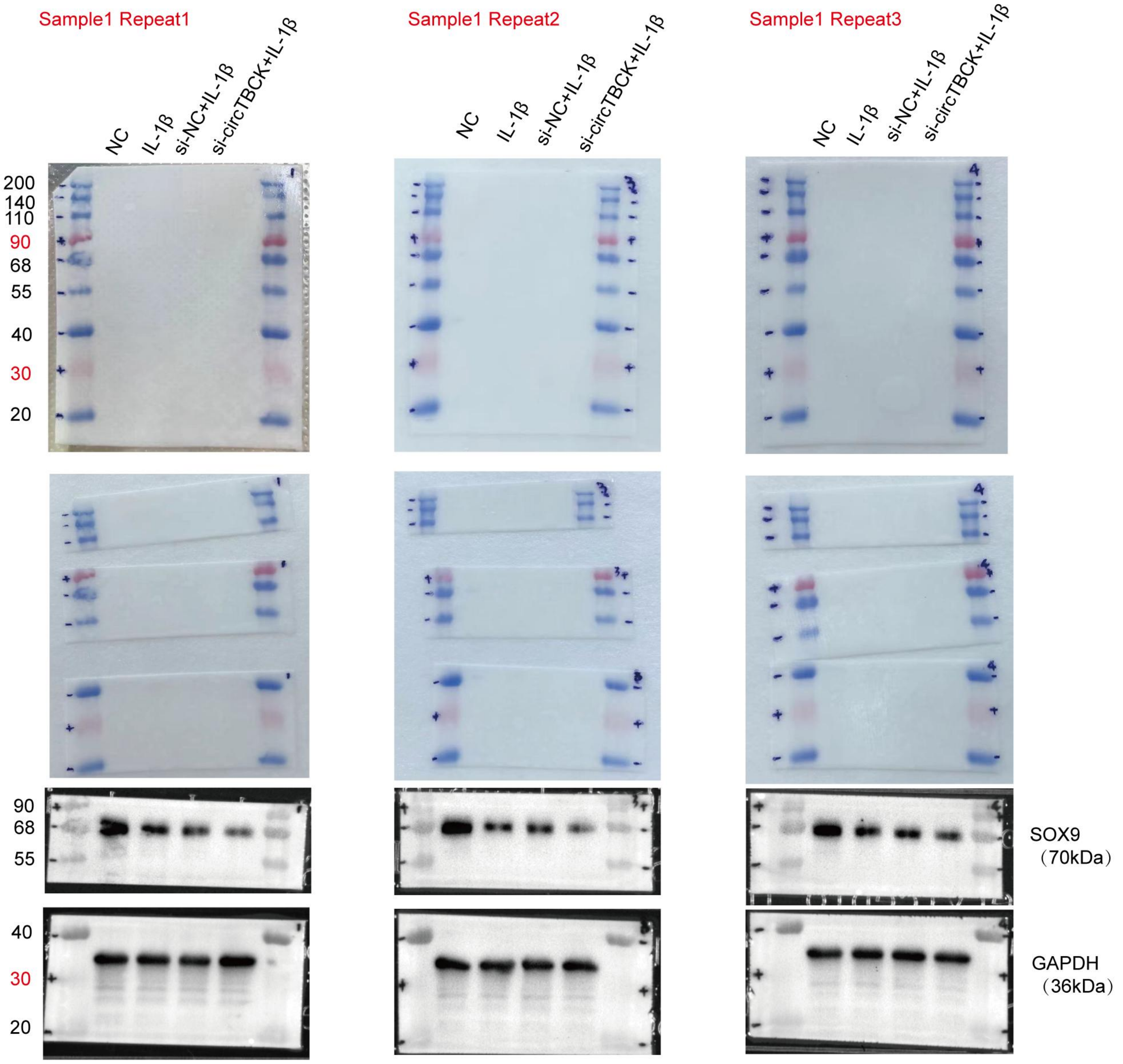

Sample1 Repeat1
Sample1 Repeat2
Sample1 Repeat3
NC
IL-1β
si-NC+IL-1β
si-circTBCK+IL-1β
200
140
110
90
68
55
40
30
20
SOX9
（70kDa）
GAPDH
（36kDa）

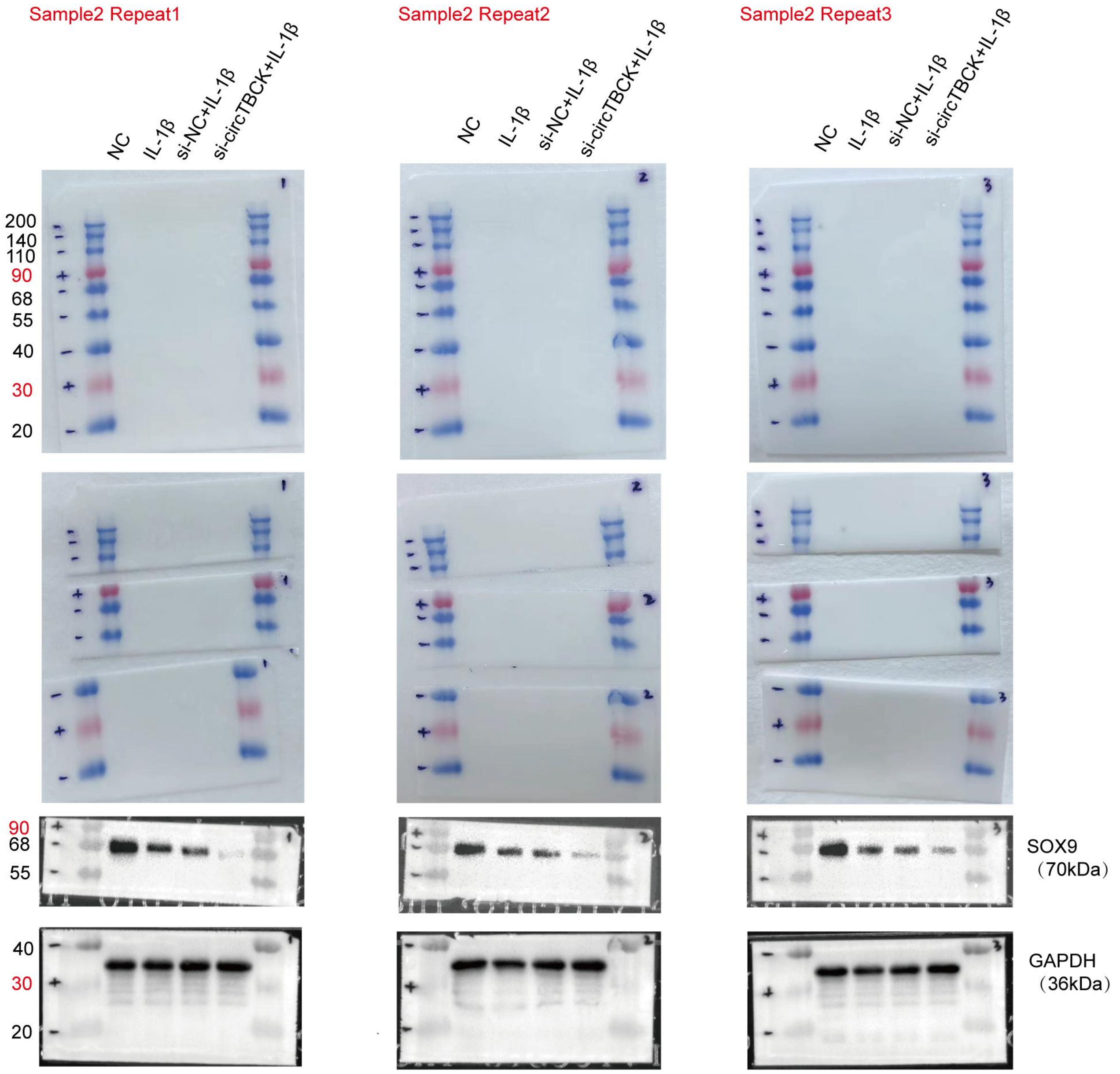
Sample2 Repeat1
Sample2 Repeat2
Sample2 Repeat3
NC
IL-1β
si-NC+IL-1β
si-circTBCK+IL-1β
200
140
110
90
68
55
40
30
20
SOX9
(70kDa)
GAPDH
(36kDa)

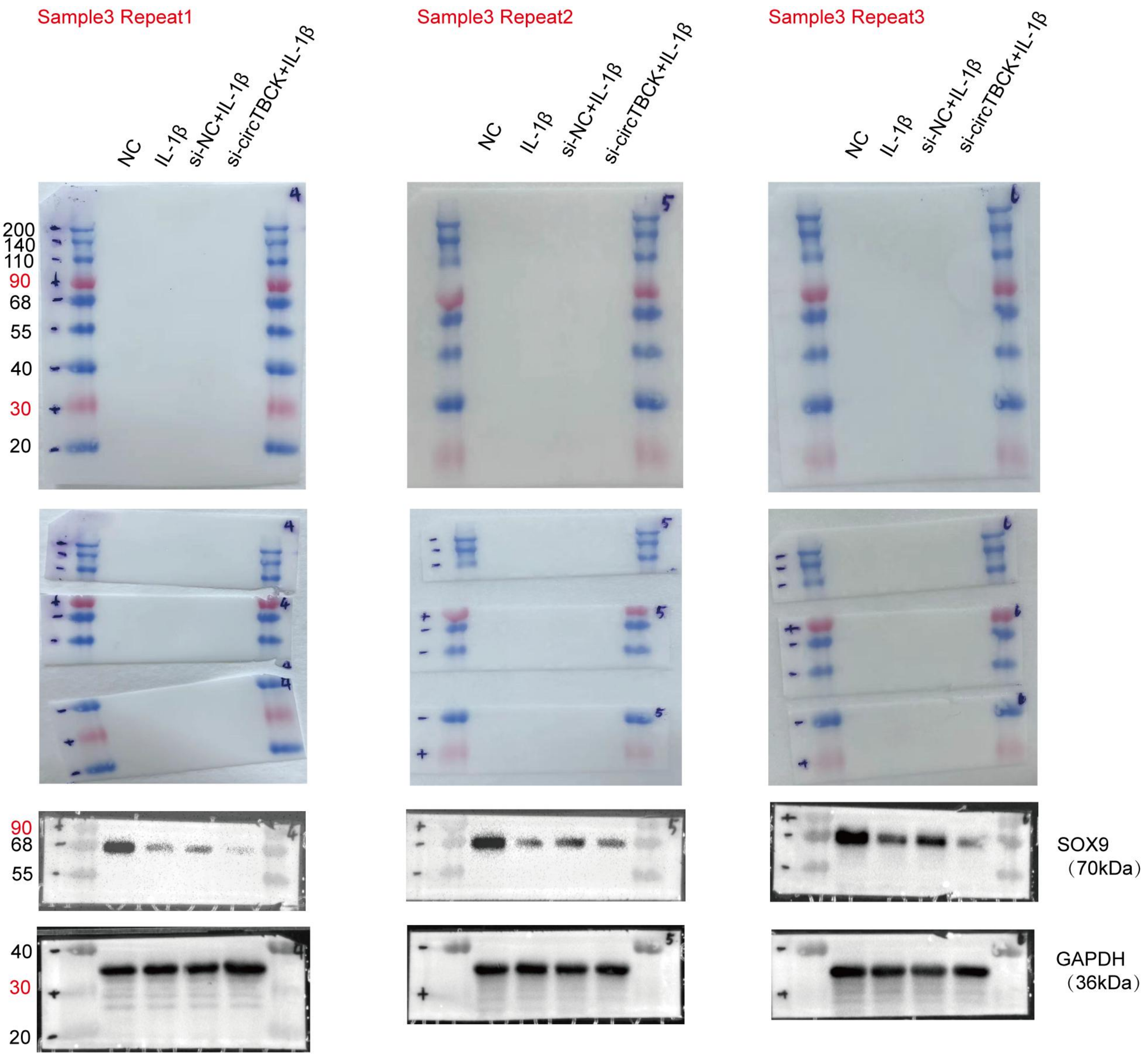
Sample3 Repeat1
Sample3 Repeat2
Sample3 Repeat3
NC
IL-1β
si-NC+IL-1β
si-circTBCK+IL-1β
200
140
110
90
68
55
40
30
20
SOX9
（70kDa）
GAPDH
（36kDa）

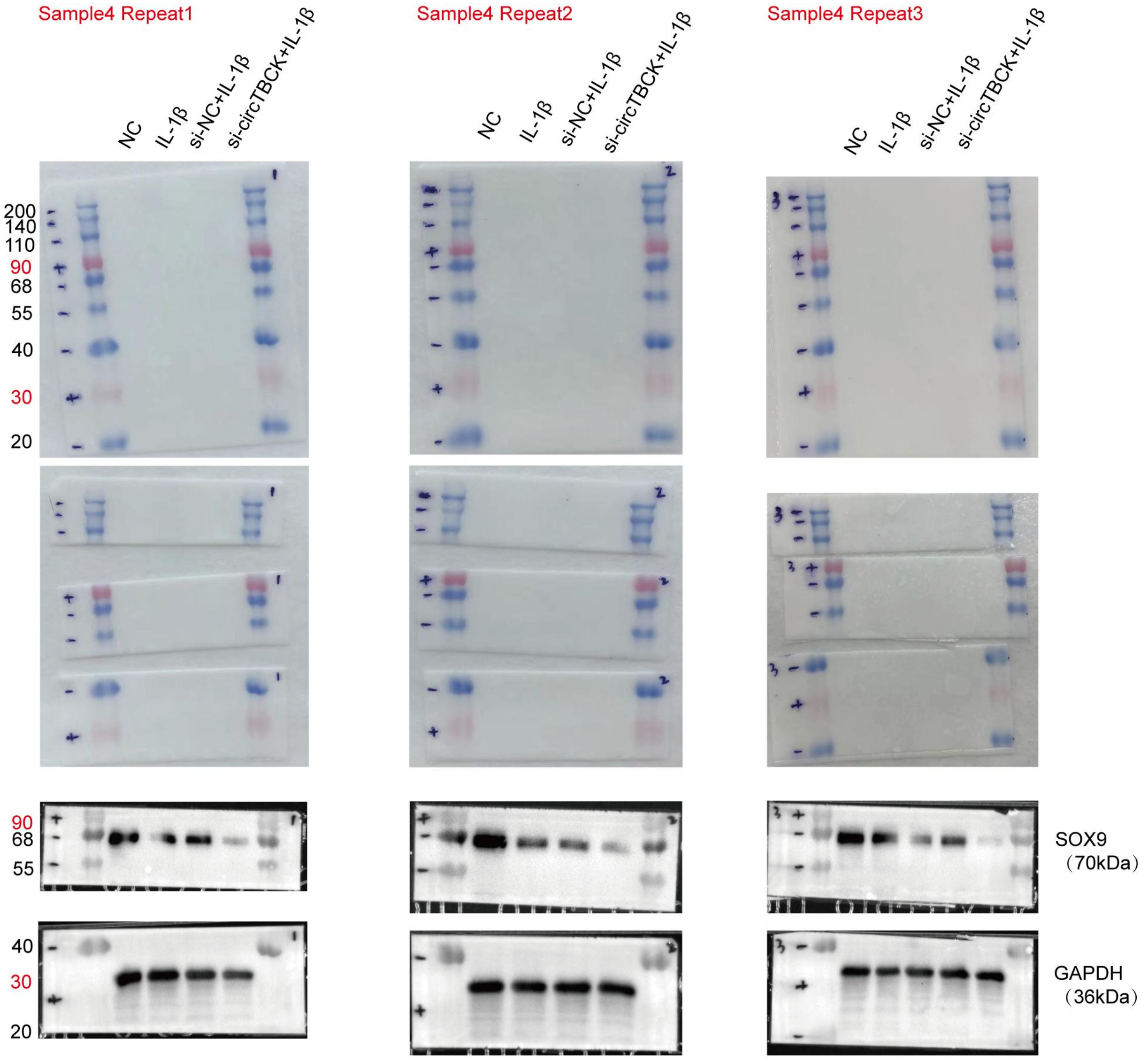

Sample4 Repeat1
Sample4 Repeat2
Sample4 Repeat3
NC
IL-1β
si-NC+IL-1β
si-circTBCK+IL-1β
200
140
110
90
68
55
40
30
20
SOX9
（70kDa）
GAPDH
（36kDa）
