# Supplementary material for: CircTBCK protects against osteoarthritis by regulating extracellular matrix and autophagy
Source: Hum Cell. 2025 Feb 25;38(2):60. doi: 10.1007/s13577-025-01186-y (PMC11860995; doi:10.1007/s13577-025-01186-y)
Supplement: Supplementary file 4 — Supplementary file4 (PDF 108 KB) [file 13577_2025_1186_MOESM4_ESM.pdf]

article title:CircTBCK protects against osteoarthritis by regulating extracellular matrix and autophagy
journal name:Human cell
author names:Wei Wang, Yuzhe Sun, Peng Tang, Rui Zhang, Yufeng Jiang, Hongwei Min, Chen Gao
affiliation address:China Rehabilitation Research Center, No. 10 Jiaomen North Road, Majiabao Street, Fengtai District, Beijing, China
e-mail address:Chen Gao: gavinice@163.com；Hongwei Min: drmhw@163.com

Supplementary Table S1:The specific primers utilized for qRT-PCR

circPIGU-F	TTGTGTTGCCAAGTCTACCTGC
circPIGU-S	TTAATGGAGTCTTTTATTGTGGTCA
circSOCS7-F	GAATGGAGCACTATAGAGGAAACCC
circSOCS7-R	AATGTCCACGTCCACAAGCG
circRNF114-F	GTGGTTTGTCCCATCTGTGCC
circRNF114-R	CCCGGATCTTAGATAGGATGAACAC
circMLLT3-F	ACAACAACCAGTCAGAGCAACCC
circMLLT3-R	TCGACCTTAAAGGACCTTGTTGC
circDTWD2-F	TCCGACTTCCCAAACAGCC
circDTWD2-R	TGCGCAACACTCTGCTTTCC
circTTLL11-F	GAGATCCAGTCCAAACGGTGAA
circTTLL11-R	GTGTTATGGAGGGTCCCAGTCA
circPRDM15-F	TCCAGTGTTCAGGGTATCAGCAA
circPRDM15-R	GTCACTGCAAACACGCCTTCA
circEXTL3-F	CATCAAGGGTGTGGAGAGAAGC
circEXTL3-R	GTAGTTGGAATTGTAGAGCCAGGAC
circZNF12-F	TATCCAGACCCGGCTGCATTA
circZNF12-R	GAGAAGGCCACAGCCACATC
circTBCK-divergent-F	GTCAATCTGTTTTGCTGGACTCCTA
circTBCK-divergent-R	AACCAAGGGATGGCGTAGAGAT
circTBCK-convergent-F	CGCCATCCCTTGGTTTCTC
circTBCK-convergent-R	GGTAAGTCAGAGAAGAGAAGGATGC
GAPDH-F	CCTCGTCCCGTAGACAAAATG
GAPDH-R	TGAGGTCAATGAAGGGGTCGT
U6-F	CTCGCTTCGGCAGCACA
U6-R	AACGCTTCACGAATTTGCGT
ACTB-F	AGCCATGTACGTAGCCATCCA
ACTB-R	TCTCCGGAGTCCATCACAATG
MALAT1-F	GGCCAGCTGCAAACATTCAA
MALAT1-R	TGCAGTGTGCCAATGTTTCG
PCNA-F	GTCGGGTGAATTTGCACGTA
PCNA-R	CTCTATGGTTACCGCCTCCTC
Ki67-F	CCATCATTGACCGCTCCTTTAG

Ki67-R	CACTCTTGTCAGGGTCAGCA
SOX9-F	TGAAGATGACCGACGAGCAG
SOX9-R	GGATGCACACGGGGAACTTA
Beclin1-F	TTTTCTGGACTGTGTGCAGC
Beclin1-R	GCTTTTGTCCACTGCTCCTC
LC3-F	TTCTTCCTCCTGGTGAATGG
LC3-R	GTCTCCTGCGAGGCATAAAC
ATG5-F	TGTGCTTCGAGATGTGTGGTT
ATG5-R	GTCAAATAGCTGACTCTTGGCAA

Supplementary Table S2:The primary antibodies used for western blotting

Antibodies for Western Blotting	Article number and brand	Working concentration
collagenII	#ab34712，abcam	1：1000
SOX9	#82630，CST	1：1000
p62	#5114，CST	1：1000
LC3	#12741T，CST	1：1000
GAPDH	#10494-1-AP，Proteintech	1：5000
