# Supplementary material for: CircTBCK protects against osteoarthritis by regulating extracellular matrix and autophagy
Source: Hum Cell. 2025 Feb 25;38(2):60. doi: 10.1007/s13577-025-01186-y (PMC11860995; doi:10.1007/s13577-025-01186-y)
Supplement: Supplementary file 6 — Supplementary file6 (PDF 646 KB) [file 13577_2025_1186_MOESM6_ESM.pdf]

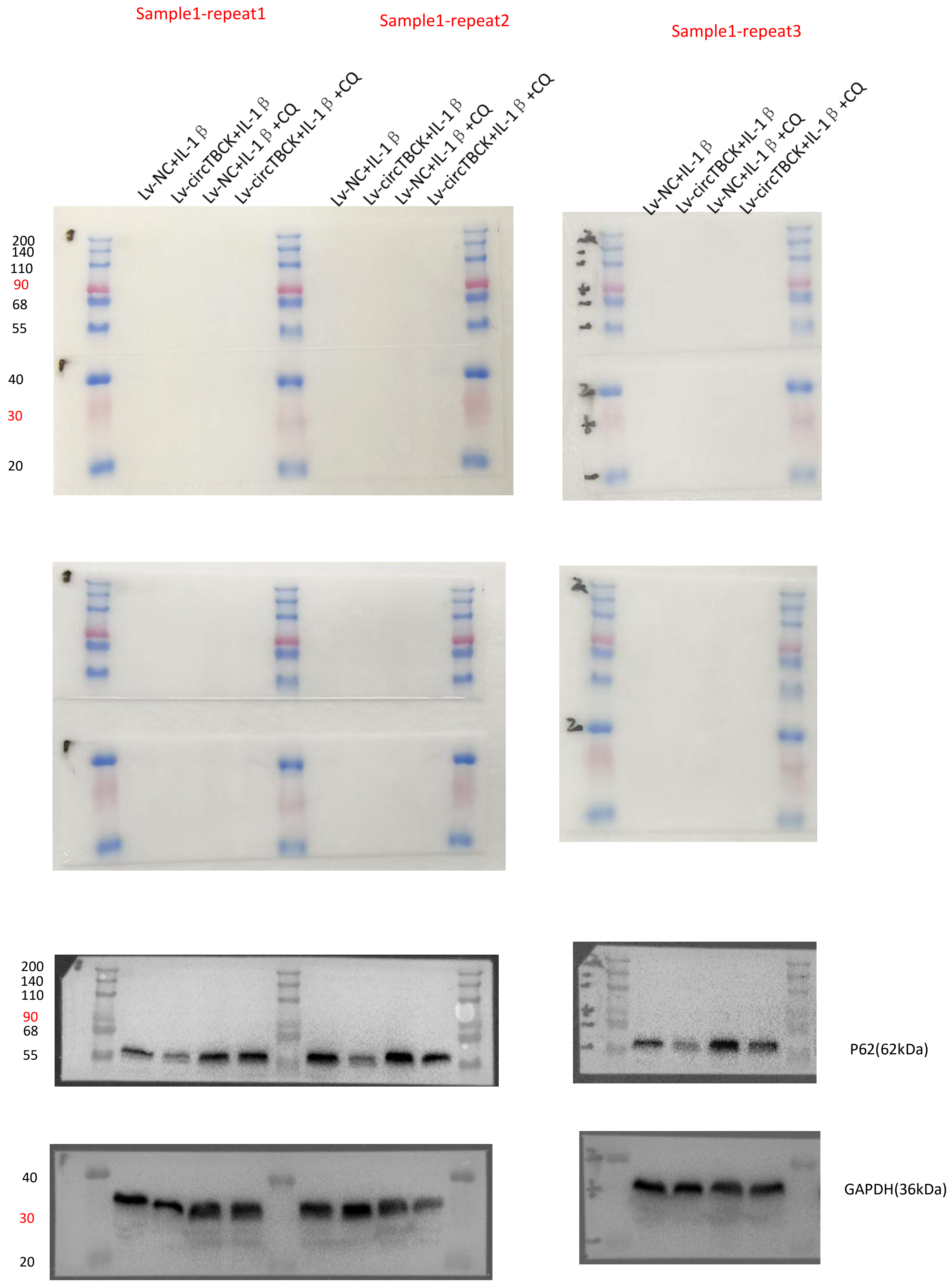

Sample1-repeat1
Sample1-repeat2
Sample1-repeat3
Lv-NC+IL-1β
Lv-circTBCK+IL-1β
Lv-NC+IL-1β+CQ
Lv-circTBCK+IL-1β+CQ
Lv-NC+IL-1β
Lv-circTBCK+IL-1β
Lv-NC+IL-1β+CQ
Lv-circTBCK+IL-1β+CQ
Lv-NC+IL-1β
Lv-circTBCK+IL-1β
Lv-NC+IL-1β+CQ
Lv-circTBCK+IL-1β+CQ
200
140
110
90
68
55
40
30
20
200
140
110
90
68
55
40
30
20
P62(62kDa)
GAPDH(36kDa)

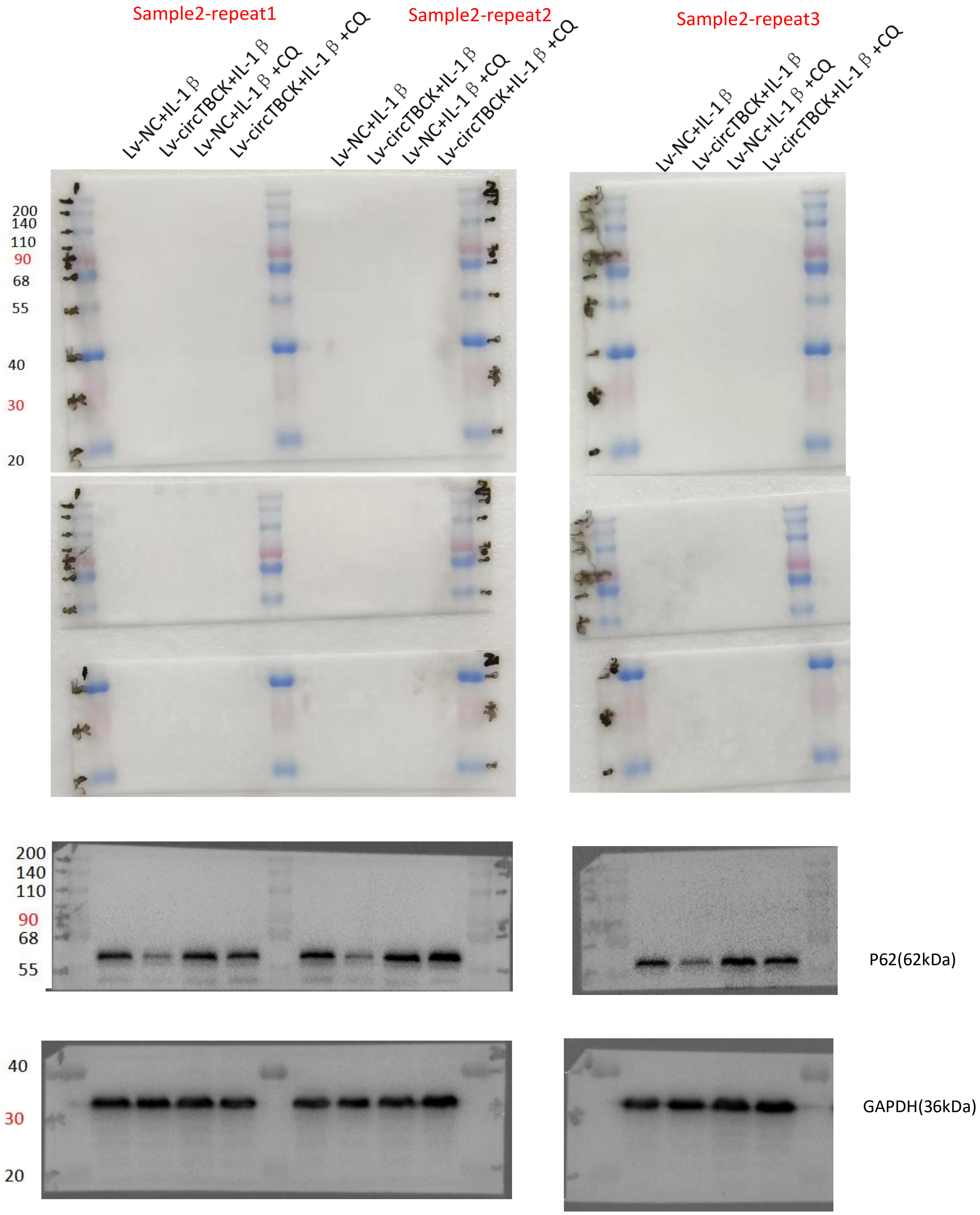
Sample2-repeat1
Sample2-repeat2
Sample2-repeat3
Lv-NC+IL-1β
Lv-circTBCK+IL-1β
Lv-NC+IL-1β+CQ
Lv-circTBCK+IL-1β+CQ
200
140
110
90
68
55
40
30
20
P62(62kDa)
GAPDH(36kDa)

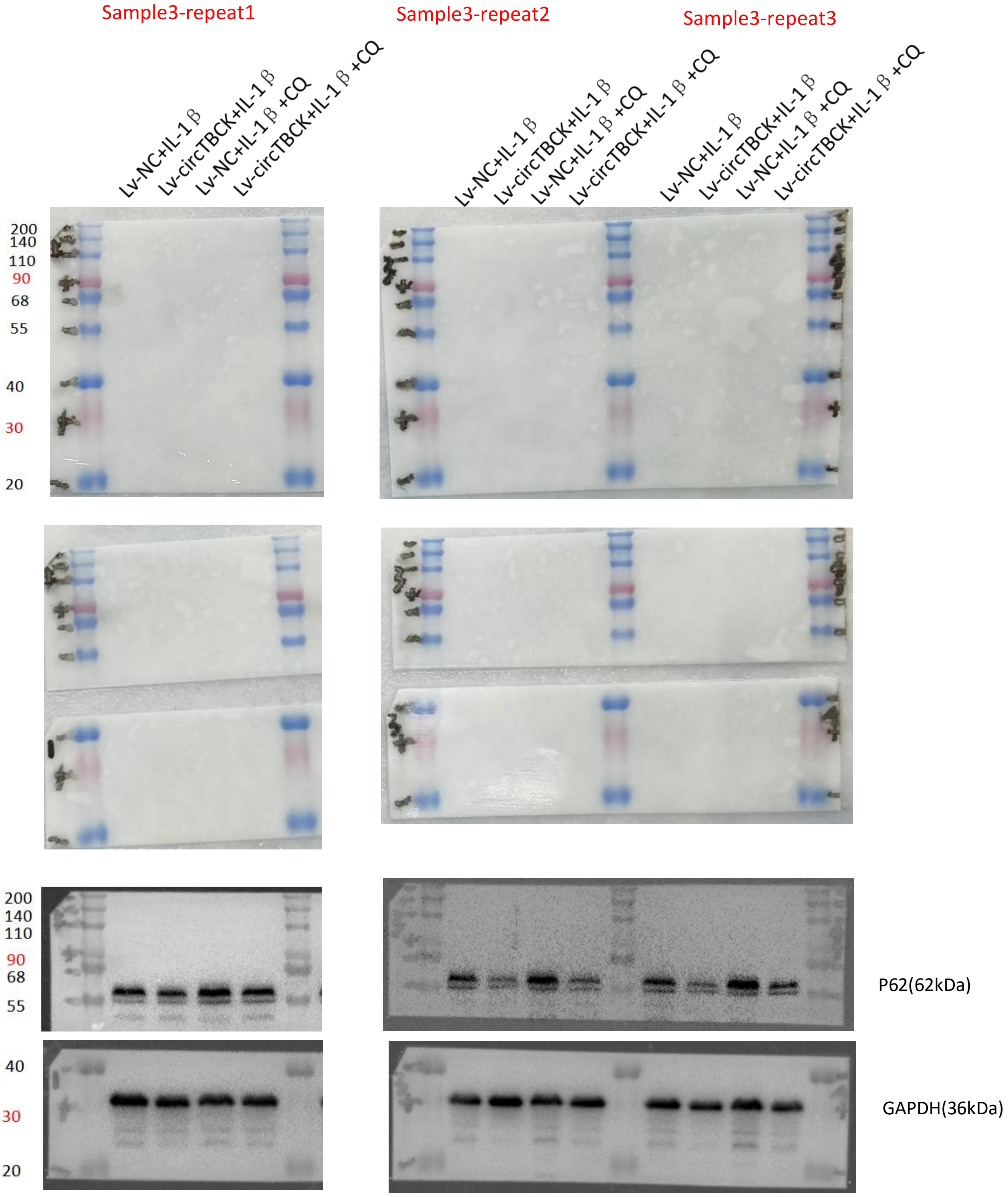
Sample3-repeat1
Sample3-repeat2
Sample3-repeat3
Lv-NC+IL-1β
Lv-circTBCK+IL-1β
Lv-NC+IL-1β+CQ
Lv-circTBCK+IL-1β+CQ
200
140
110
90
68
55
40
30
20
P62(62kDa)
GAPDH(36kDa)
