# Supplementary material for: CircTBCK protects against osteoarthritis by regulating extracellular matrix and autophagy
Source: Hum Cell. 2025 Feb 25;38(2):60. doi: 10.1007/s13577-025-01186-y (PMC11860995; doi:10.1007/s13577-025-01186-y)
Supplement: Supplementary file 8 — Supplementary file8 (PDF 1009 KB) [file 13577_2025_1186_MOESM8_ESM.pdf]

Sample1 Repeat1

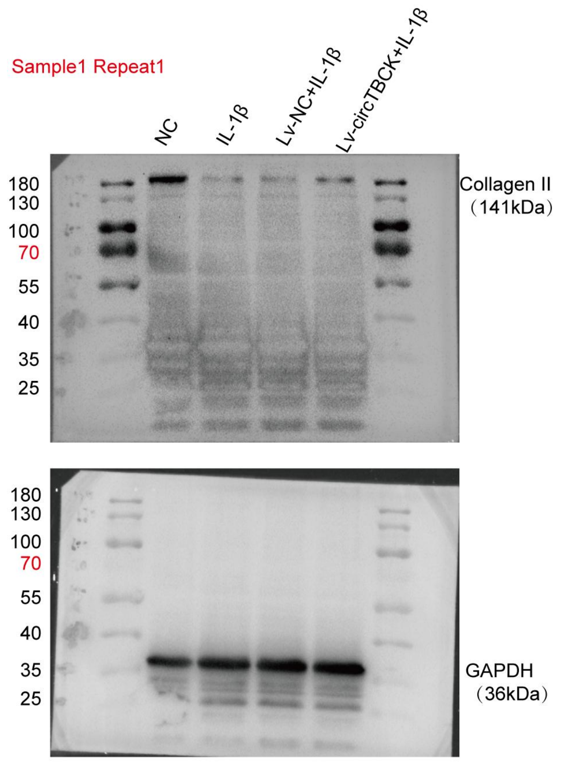

Sample1 Repeat2+3

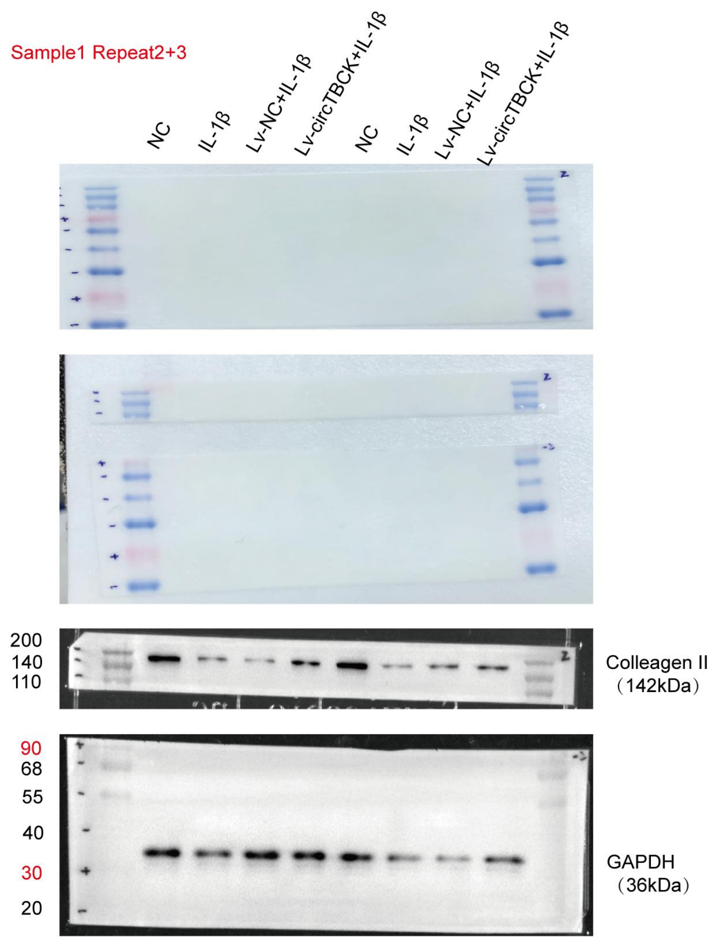

Sample2 Repeat1+2

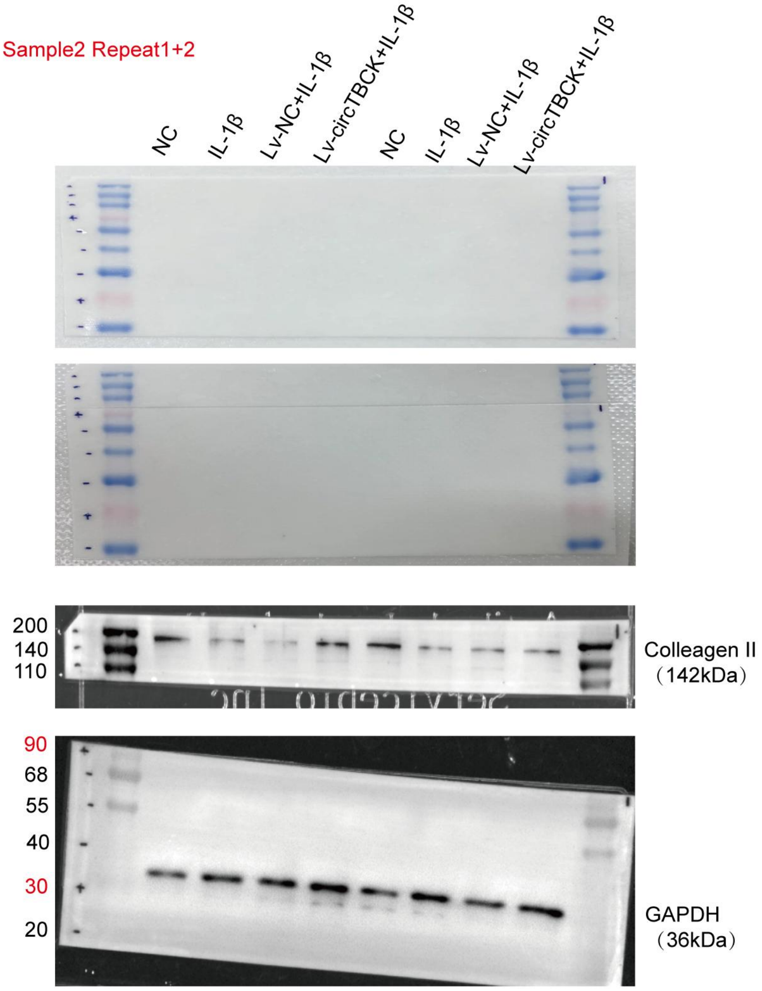

Sample2 Repeat3

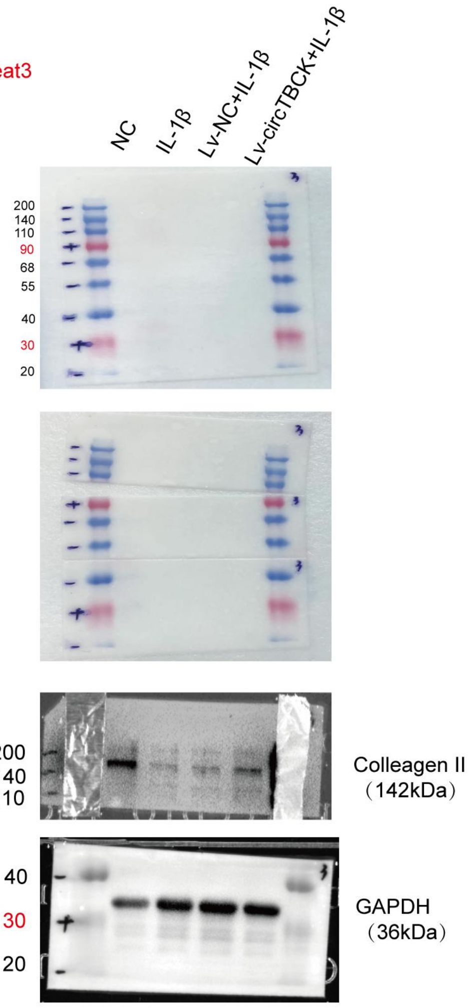

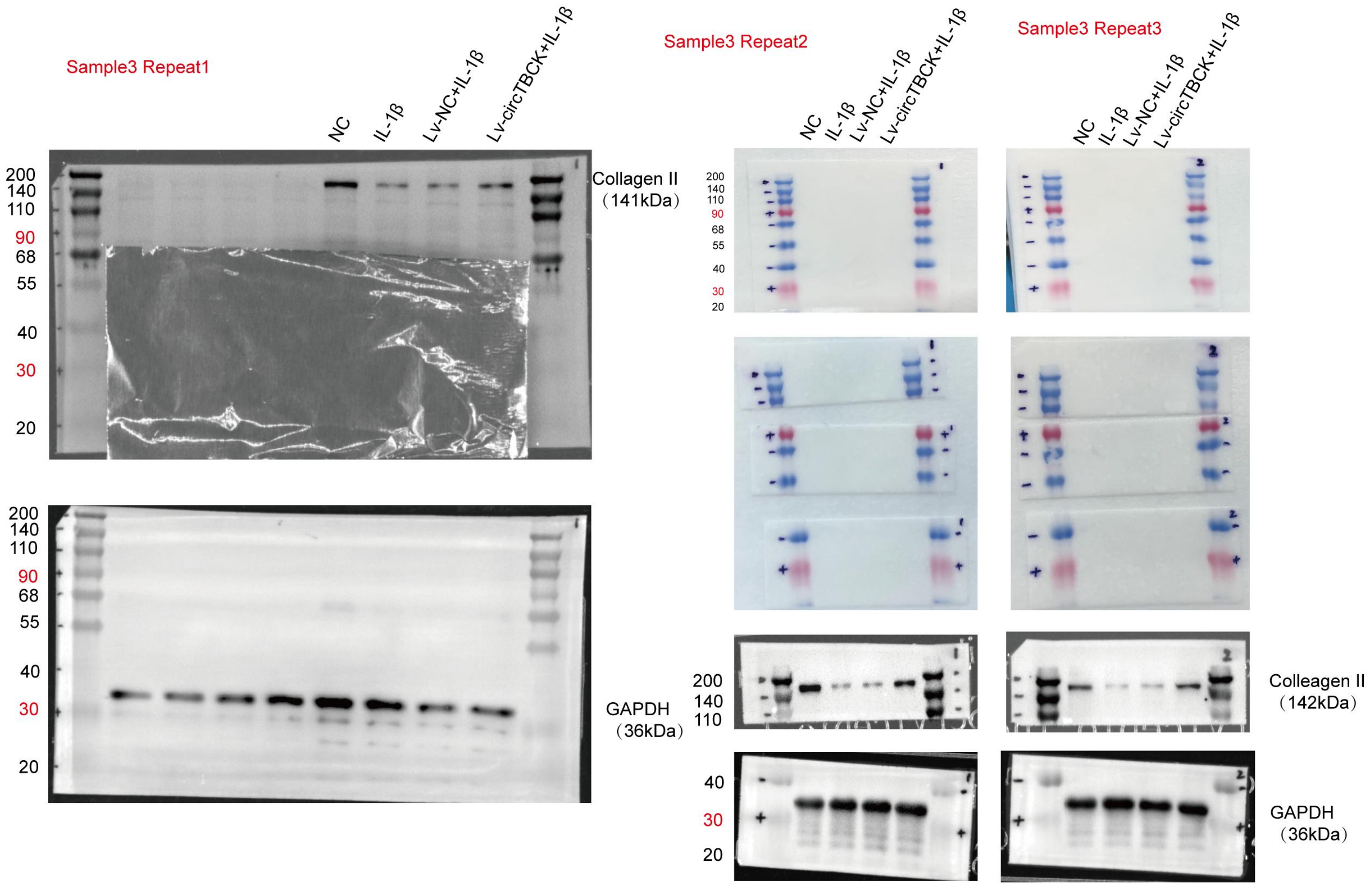
Sample3 Repeat1
NC
IL-1β
Lv-NC+IL-1β
Lv-circTBCK+IL-1β
200
140
110
90
68
55
40
30
20
Collagen II
（141kDa）
GAPDH
（36kDa）
Sample3 Repeat2
Sample3 Repeat3
Colleagen II
（142kDa）
GAPDH
（36kDa）

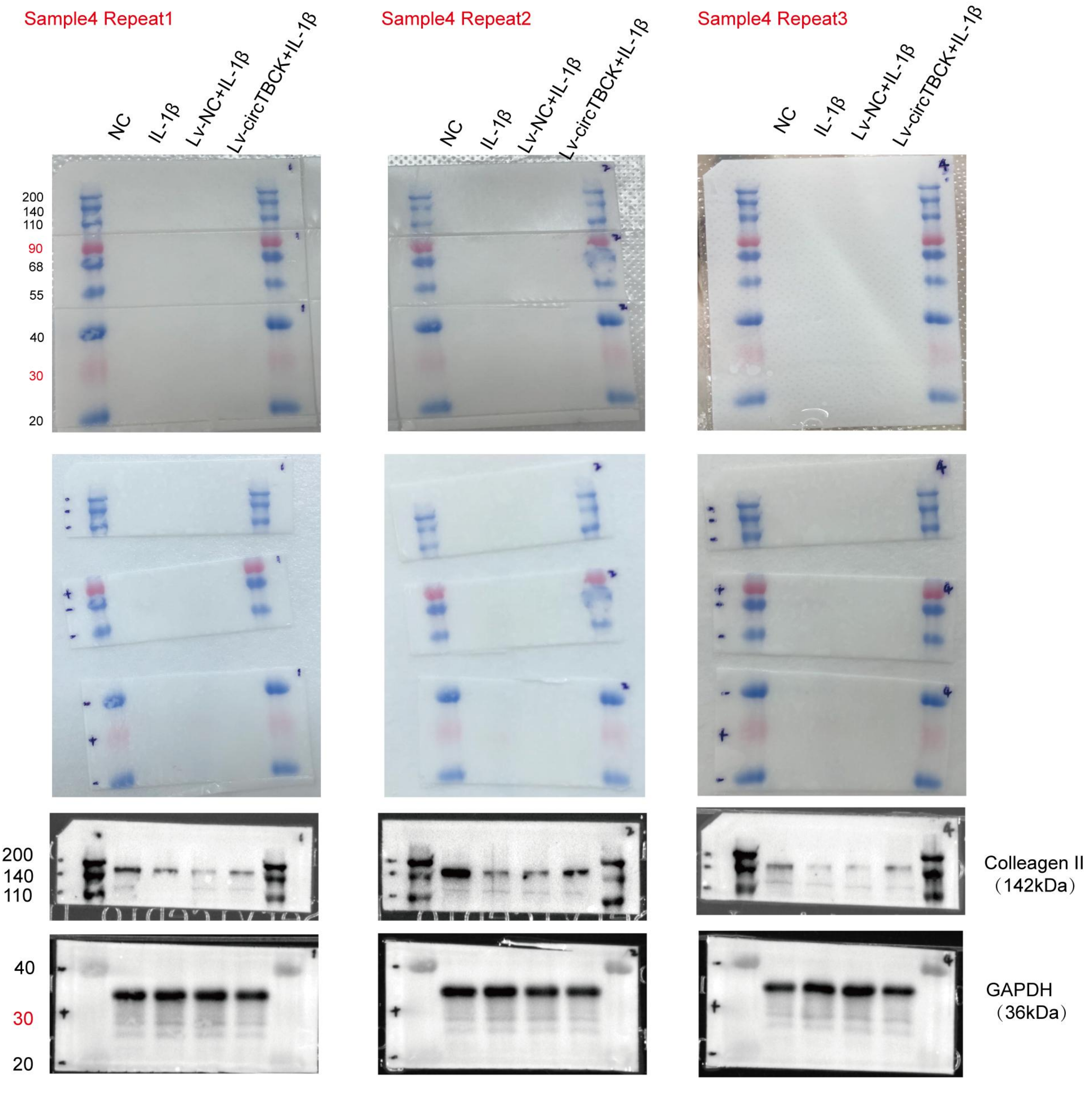
Sample4 Repeat1
Sample4 Repeat2
Sample4 Repeat3
NC
IL-1β
Lv-NC+IL-1β
Lv-circTBCK+IL-1β
200
140
110
90
68
55
40
30
20
Colleagen II
（142kDa）
GAPDH
（36kDa）
