# Supplementary material for: CircTBCK protects against osteoarthritis by regulating extracellular matrix and autophagy
Source: Hum Cell. 2025 Feb 25;38(2):60. doi: 10.1007/s13577-025-01186-y (PMC11860995; doi:10.1007/s13577-025-01186-y)
Supplement: Supplementary file 9 — Supplementary file9 (PDF 659 KB) [file 13577_2025_1186_MOESM9_ESM.pdf]

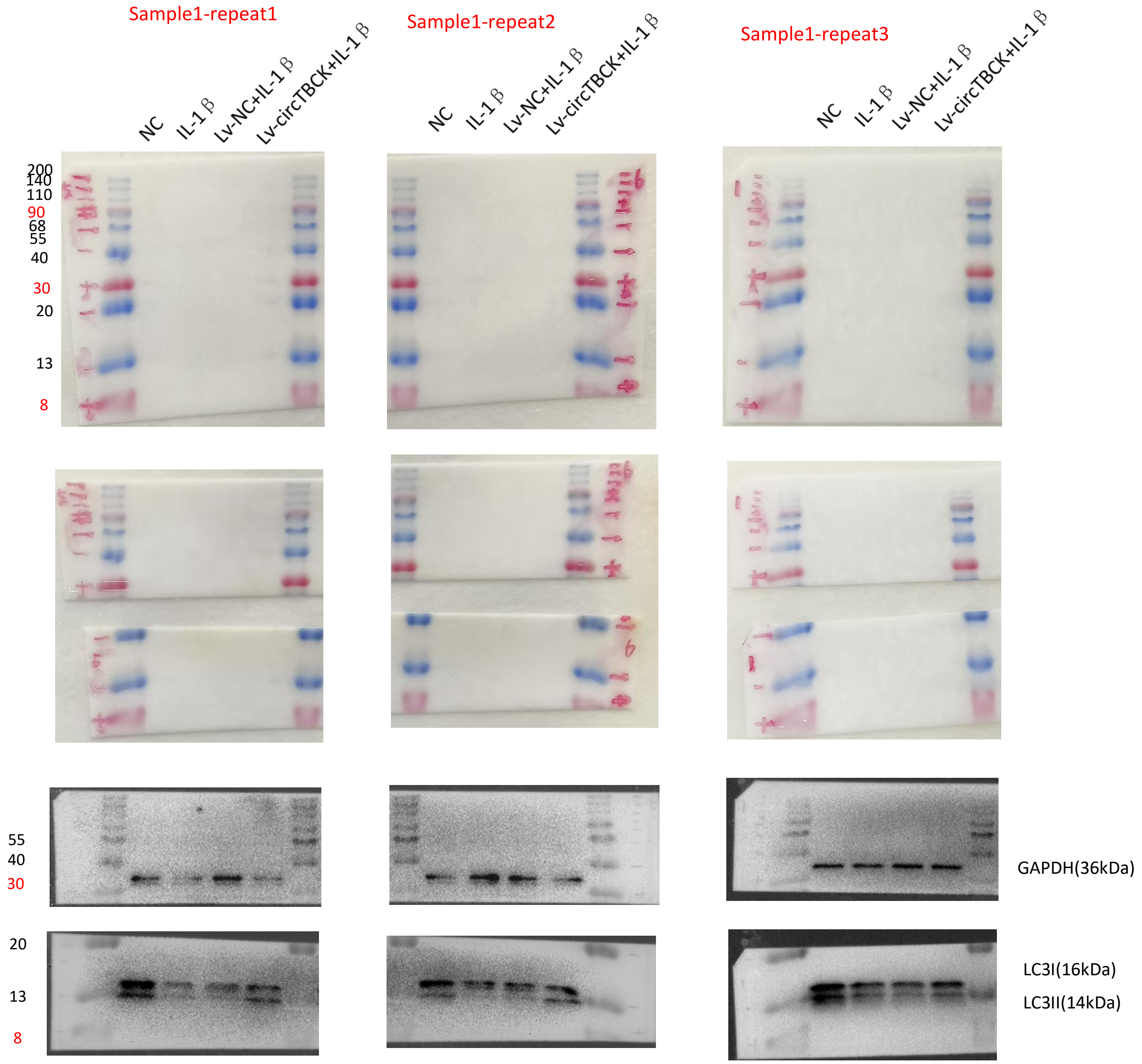
Sample1-repeat1
Sample1-repeat2
Sample1-repeat3
NC
IL-1β
Lv-NC+IL-1β
Lv-circTBCK+IL-1β
200
140
110
90
68
55
40
30
20
13
8
55
40
30
20
13
8
GAPDH(36kDa)
LC3I(16kDa)
LC3II(14kDa)

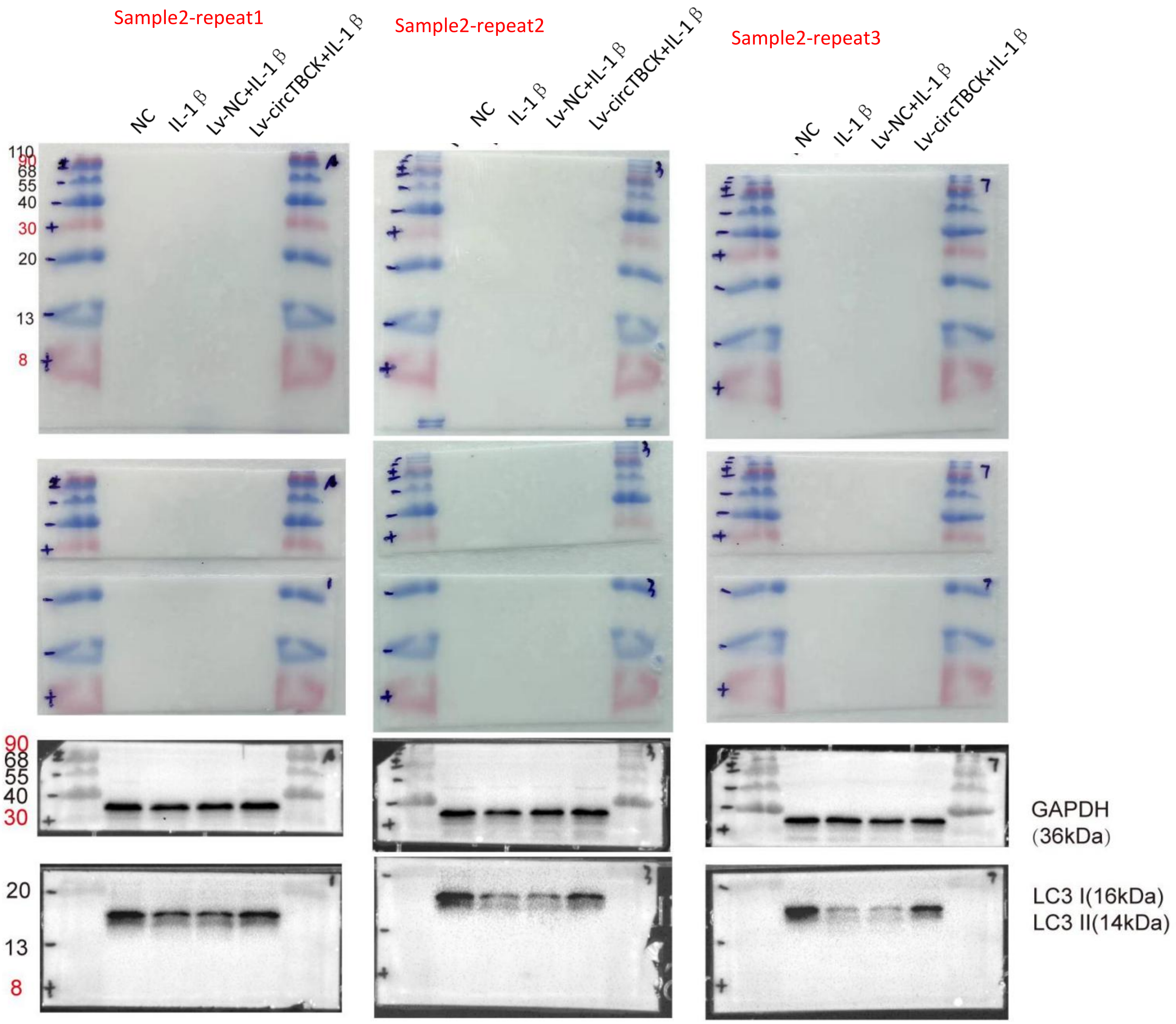

Sample2-repeat1
Sample2-repeat2
Sample2-repeat3
NC
IL-1β
Lv-NC+IL-1β
Lv-circTBCK+IL-1β
110
90
68
55
40
30
20
13
8
GAPDH
(36kDa)
LC3 I(16kDa)
LC3 II(14kDa)

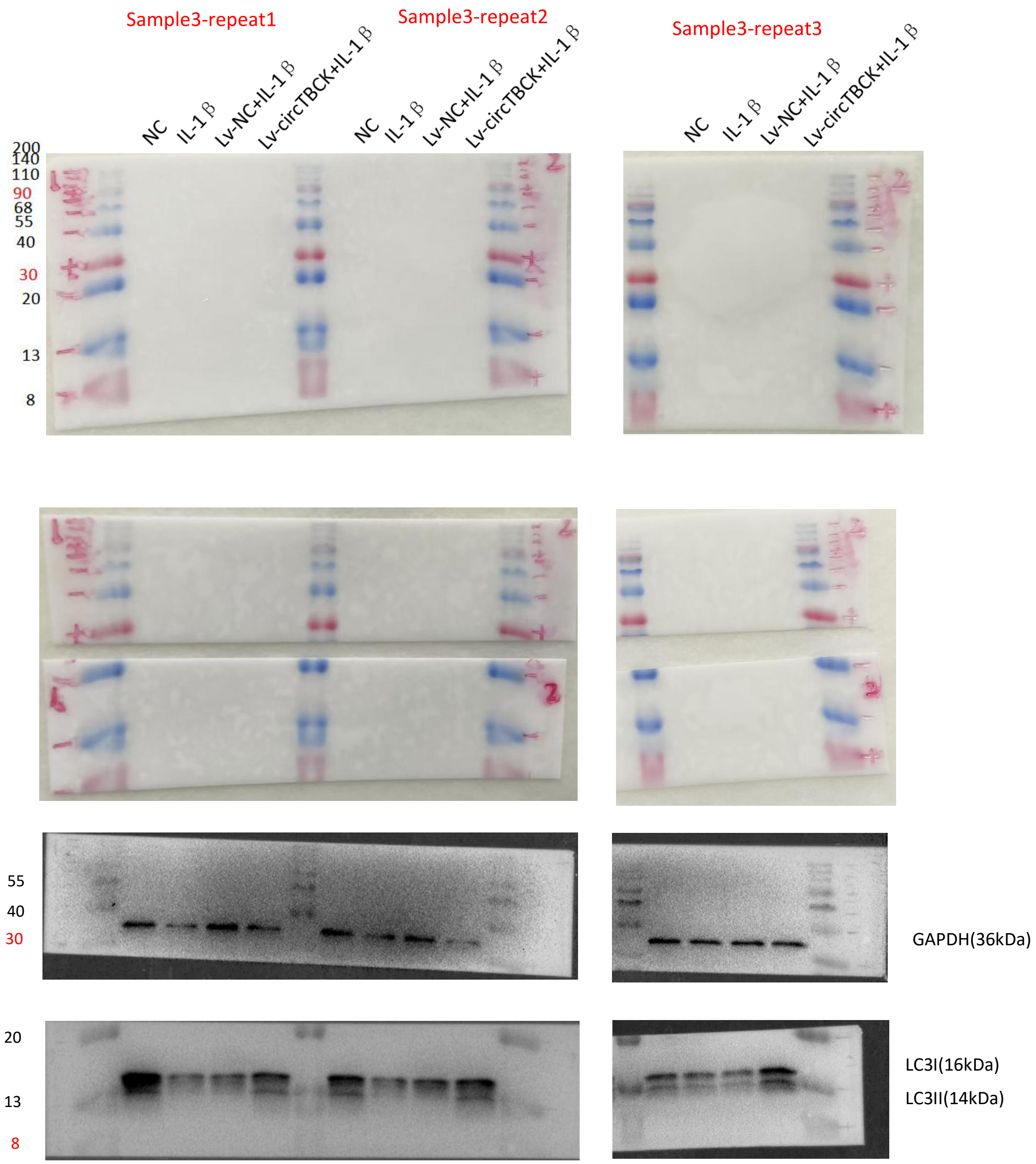

Sample3-repeat1
Sample3-repeat2
Sample3-repeat3
NC
IL-1β
Lv-NC+IL-1β
Lv-circTBCK+IL-1β
NC
IL-1β
Lv-NC+IL-1β
Lv-circTBCK+IL-1β
NC
IL-1β
Lv-NC+IL-1β
Lv-circTBCK+IL-1β
200
140
110
90
68
55
40
30
20
13
8
55
40
30
GAPDH(36kDa)
20
13
8
LC3I(16kDa)
LC3II(14kDa)
