# Supplementary figures and images for: CircTBCK protects against osteoarthritis by regulating extracellular matrix and autophagy
Source: Hum Cell. 2025 Feb 25;38(2):60. doi: 10.1007/s13577-025-01186-y (PMC11860995; doi:10.1007/s13577-025-01186-y)

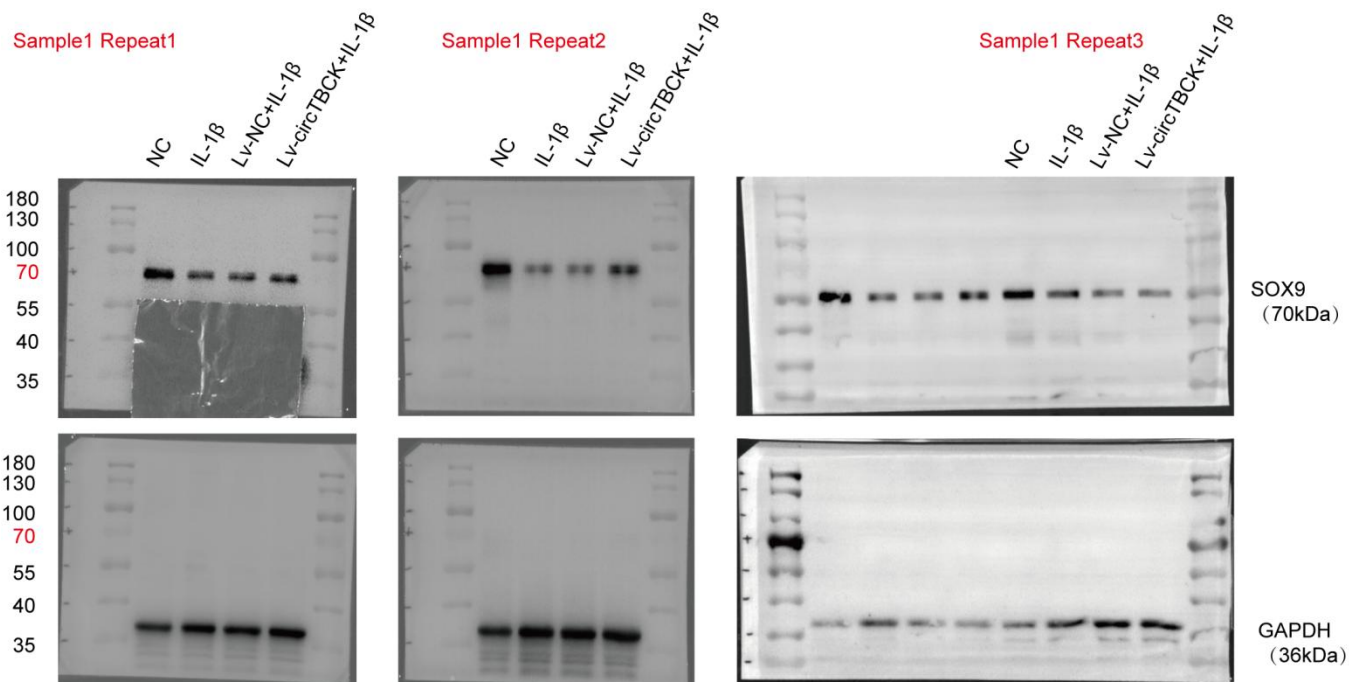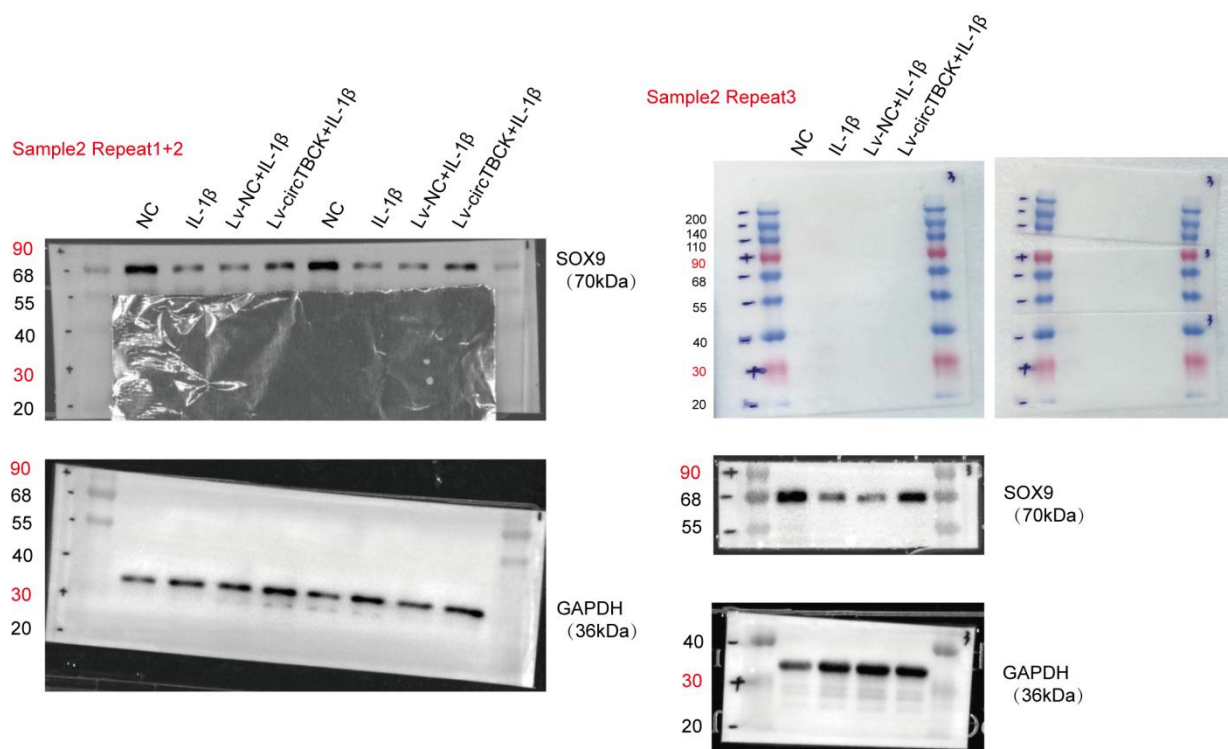

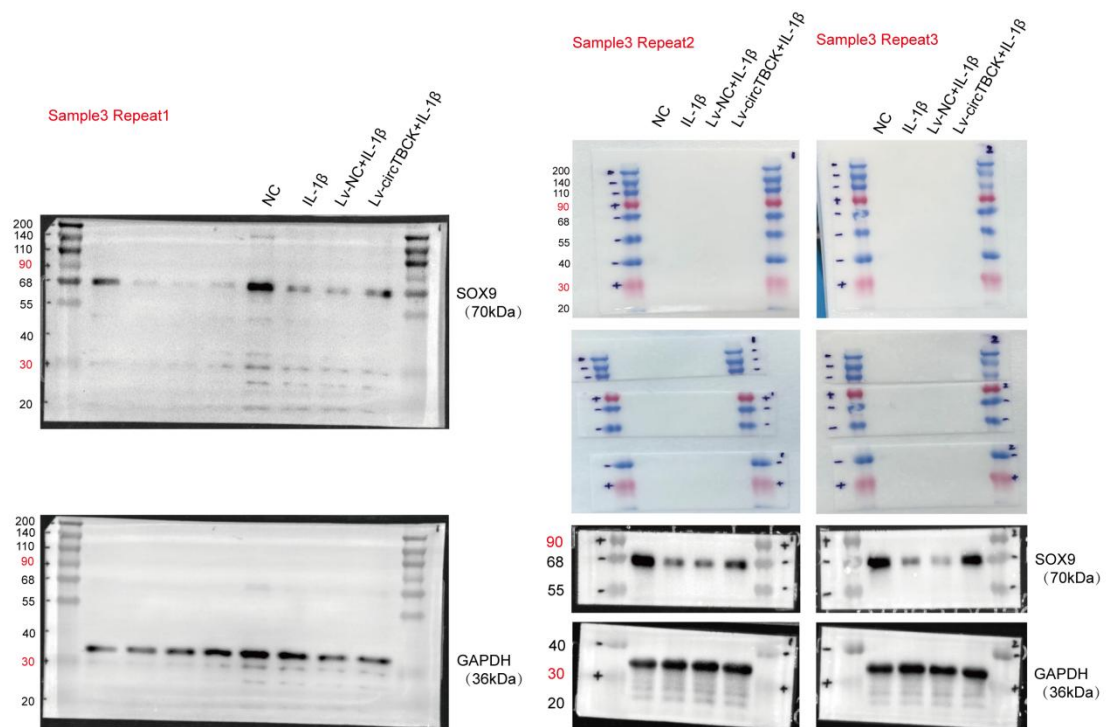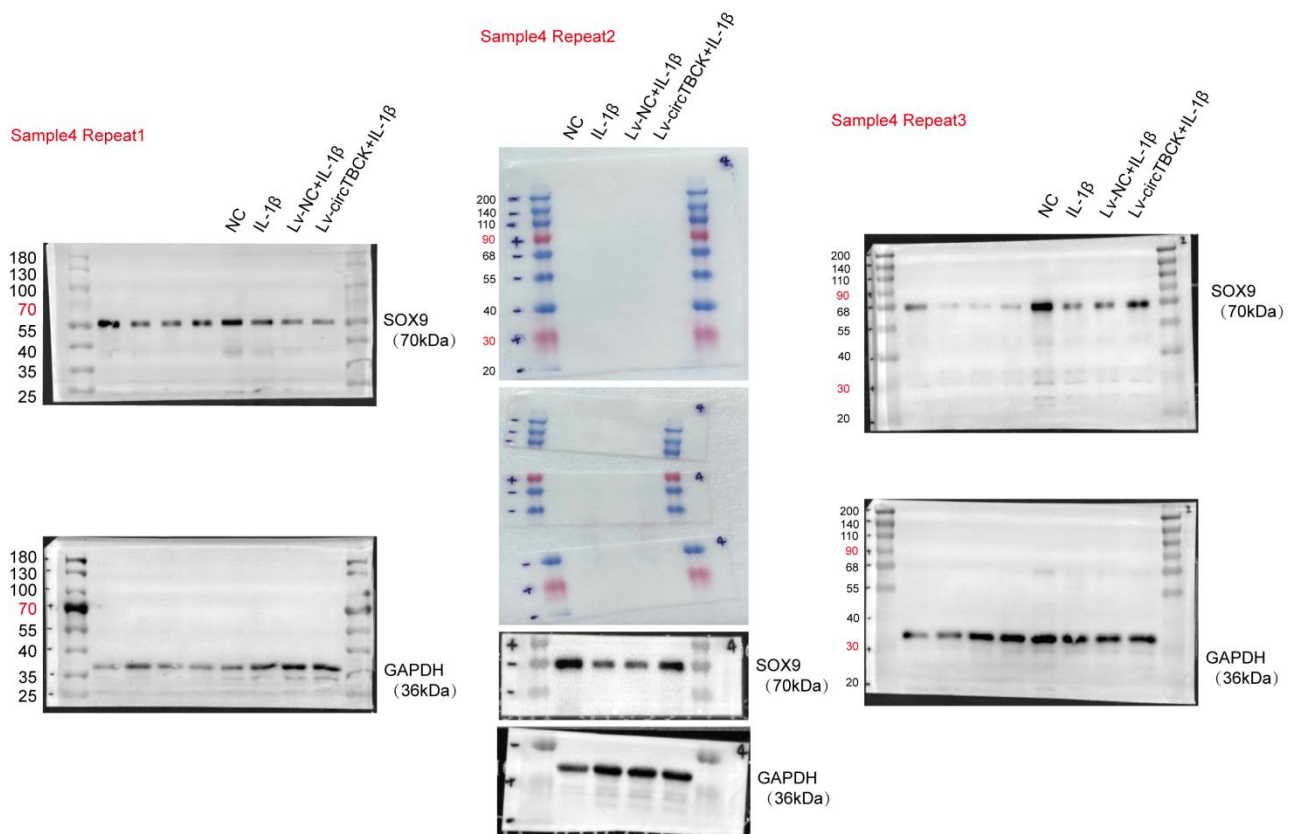

Supplement: Supplementary file 11 — Supplementary file11 (PDF 381 KB) [file 13577_2025_1186_MOESM11_ESM.pdf]
